# Sparse Functional Identification of Complex Cells from Spike Times and the Decoding of Visual Stimuli

**DOI:** 10.1186/s13408-017-0057-1

**Published:** 2018-01-18

**Authors:** Aurel A. Lazar, Nikul H. Ukani, Yiyin Zhou

**Affiliations:** Department of Electrical Engineering, Columbia University, 500 W 120th Street, Mudd 1300, New York, NY 10027 USA

**Keywords:** Encoding of visual stimuli, Complex cells, Quadratic receptive fields, Dendritic stimulus processors, Sparse neural decoding, Sparse functional identification, Duality between decoding and functional identification

## Abstract

We investigate the sparse functional identification of complex cells and the decoding of spatio-temporal visual stimuli encoded by an ensemble of complex cells. The reconstruction algorithm is formulated as a rank minimization problem that significantly reduces the number of sampling measurements (spikes) required for decoding. We also establish the duality between sparse decoding and functional identification and provide algorithms for identification of low-rank dendritic stimulus processors. The duality enables us to efficiently evaluate our functional identification algorithms by reconstructing novel stimuli in the input space. Finally, we demonstrate that our identification algorithms substantially outperform the generalized quadratic model, the nonlinear input model, and the widely used spike-triggered covariance algorithm.

**Electronic Supplementary Material:**

The online version of this article (10.1186/s13408-017-0057-1) contains supplementary material.

## Introduction

**Fig. 1:**
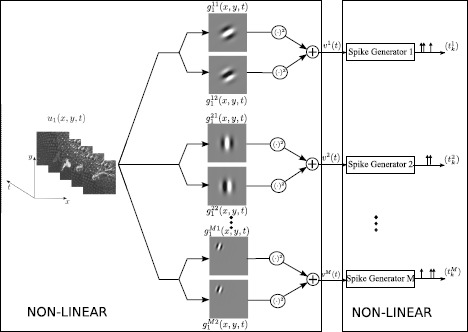
A neural circuit consisting of a population of complex cells

It is widely accepted that the early mammalian visual system employs a series of neural circuits to extract elementary visual features, such as edges and motion [1, 2]. Feature extraction capabilities of simple and complex cells arising in the primary visual cortex (V1) have been extensively investigated. Layer IV simple cells receive direct input from the Lateral Geniculate Nucleus [3]. Each simple cell consists of a linear receptive field cascaded with a highly nonlinear spike generator. Complex cells in layer II/III of V1 sum the output of a pool of simple cells having similar orientation selectivity and spatial extent [4] and are thereby selective to oriented edges/lines over a spatially restricted region of the visual field [1]. Whereas simple cells respond maximally to a particular phase of the edge, complex cells are largely phase invariant [5, 6]. Therefore, the receptive fields of complex cells cannot be simply mapped into excitatory and inhibitory regions [1]. Receptive fields of simple cells are often modeled as spatio-temporal linear filters with a spatial impulse response that resemble Gabor functions [7], whereas the receptive fields of complex cells are often modeled as sums of squared linear filters [8]. For simplicity, a quadrature pair of space-time Gabor filters has been employed in an energy model of complex cells [9–11]. Neural circuits comprising complex cells constitute a highly nonlinear circuit as illustrated in Fig. 1.

Feedforward projections from V1 to other cortical areas mainly originate from layer II/III [12], suggesting that complex cells play a critical role in relaying visual information processed in V1 to higher brain areas. Whereas tuning properties of individual complex cells have been characterized [13, 14], the information about visual stimuli that an ensemble of complex cells can provide and how efficiently they can represent such information has yet to be elucidated.

Under the modeling framework of time encoding machines (TEMs) [15, 16], it has been shown that decoding of stimuli and functional identification of linear receptive fields of simple cells are dual to each other [17]. This led to mathematically rigorous identification algorithms for identifying linear receptive fields of simple cells [17]. By modeling the nonlinear processing in complex cells as Volterra dendritic stimulus processors (DSPs) [18, 19], the representation of stimuli encoded by spike times generated by neural circuits with complex cells was also exhaustively analyzed. Functional identification of a complex cell DSP was possible again thanks to the demonstrated duality between decoding and functional identification. Although these theoretical methods exhibit deep structural properties, they have been shown to be tractable only for decoding and functional identification problems of small dimensions. In their current form, they are not tractable due to the ‘curse of dimensionality’ [20].

The nonlinear transformations taking place in the DSP of complex cells lead to loss of phase information. Previous work has empirically found that static images recovered from the magnitude response of Gabor wavelets are perceptually recognizable, albeit they exhibit significant errors in their pixel intensity values [21]. With this in mind, we formulate the reconstruction of stimuli encoded with complex cells as a phase retrieval problem [22] and, in search of tractable algorithms, utilize recent developments in optimization theory of low-rank matrices [22–24]. Applying such methods, we develop algorithms that are highly effective in decoding visual stimuli encoded by complex cells. As will be detailed in the next sections, the complex cells, as defined in this paper, have DSP kernels that are low-rank and include those shown in Fig. 1 as a particular case.

After demonstrating that the decoding of visual stimuli becomes tractable, we describe sparse algorithms that functionally identify the DSPs of complex cells using the spike times they generate. The sparse identification algorithms are based on the key observation that functional identification can be viewed as the dual problem of decoding stimuli that are encoded by an ensemble of complex cells. Although a generalization of the duality results from simple cells to complex cells was already given in [18], we show in this paper that these results remain valid under the assumption of sparsity, that is, for the case of low-rank DSP kernels. This significantly reduces the time of stimulus presentation that is needed in the identification process. The sparse duality result also enables us to evaluate the identified circuits in the input space. We achieve the latter by computing the mean square error or signal-to-noise ratio (SNR) of novel stimuli decoded using the identified circuits [17]. The sparse decoding and functional identification algorithms presented here apply to circuits build around a wide range of neuron models including integrate-and-fire neurons with random thresholds and biophysically realistic conductance-based models with intrinsic noise.

This paper is organized as follows. In Sect. 2, we first introduce the modeling of encoding of temporal stimuli with complex cells. We provide a detailed review of decoding of stimuli and the functional identification of complex cells and point out the current algorithmic limitations. In Sect. 3, we provide sparse decoding algorithms that achieve high accuracy and are algorithmically tractable. We then explicate the dual relationship between sparse functional identification and decoding and provide examples for the identification of low-rank temporal DSP kernels of complex cells. In Sect. 4, we extend sparse decoding methodology to spatio-temporal stimuli and functional identification of spatio-temporal complex cells. Using novel stimuli, we provide evaluation examples of the identification algorithms in the input space and compare them with other state-of-the-art methods. Finally, we conclude in Sect. 5 and suggest how the approach advanced in this paper can be applied beyond complex cells.

## Neural Circuits with Complex Cells: Encoding, Decoding, and Functional Identification

In this section, we model the encoding of temporal stimuli by a neural circuit consisting of neurons akin to complex cells. We start by modeling the space of temporal stimuli in Sect. 2.1. In Sect. 2.2, the model of encoding is formally described. In Sect. 2.3, we proceed to present a reconstruction algorithm for decoding temporal stimuli encoded by the neural circuit. A method for functional identification of neurons constituting the neural circuit is provided in Sect. 2.4. The reconstruction algorithm and the functional identification algorithm discussed in this section are based on [18].

### Modeling Temporal Stimuli

We model the temporal varying stimuli $u_{1}=u_{1}(t)$, $t \in \mathbb{D}$, as real-valued elements of the space of trigonometric polynomials [15]. The choice of the space of the trigonometric polynomials has, as we will see, substantial computational advantages.

#### Definition 1

The space of trigonometric polynomials ${\mathcal {H}}_{1}$ is the Hilbert space of complex-valued functions

1 $$ u_{1}(t) = \sum_{l_{t}=-L_{t}}^{L_{t}} c_{l_{t}}e_{l_{t}}(t) $$

over the domain $\mathbb{D} = [0, S_{t}]$, where

$$ e_{l_{t}}(t) = \frac{1}{\sqrt{S_{t}}}\operatorname{exp} \biggl( \frac{jl _{t}\varOmega_{t}}{L_{t}}t \biggr) , $$

and $c_{l_{t}}$, $l_{t}=-L_{t},\ldots,L_{t}$, are the coefficients of $u_{1}$ in ${\mathcal {H}}_{1}$. Here $\varOmega_{t}$ denotes the bandwidth, and $L_{t}$ is the order of the space. Stimuli $u_{1}\in {\mathcal {H}}_{1}$ are extended to be periodic over ${\mathbb {R}}$ with period $S_{t} = 2\pi L_{t}$/$\varOmega_{t}$.

We denote the dimension of ${\mathcal {H}}_{1}$ by $\operatorname{dim}({\mathcal {H}}_{1})$, and $\operatorname{dim}({\mathcal {H}}_{1}) = 2L_{t}+1$.

#### Definition 2

The tensor product space ${\mathcal {H}}_{2} = {\mathcal {H}}_{1} \otimes {\mathcal {H}}_{1}$ is the Hilbert space of complex-valued functions

2 $$ u_{2}(t_{1};t_{2}) = \sum _{l_{t_{1}}=-L_{t}}^{L_{t}}\sum_{l_{t_{2}}=-L_{t}}^{L_{t}} d_{l _{t_{1}} l_{t_{2}}} e_{l_{t_{1}}}(t_{1}) \cdot e_{l_{t_{2}}}(t_{2}) $$

over the domain $\mathbb{D}^{2} = [0, S_{t}] \times [0, S_{t}]$, where $d_{l_{t_{1}} l_{t_{2}}}$, $l_{t_{1}} l_{t_{2}} \in \mathbb{D}^{2}$, are the coefficients of $u_{2}$ in ${\mathcal {H}}_{2}$.

Note that $\operatorname{dim}({\mathcal {H}}_{2}) = \operatorname{dim}({\mathcal {H}}_{1})^{2}$.

### Encoding of Temporal Stimuli by a Population of Complex Cells

**Fig. 2:**
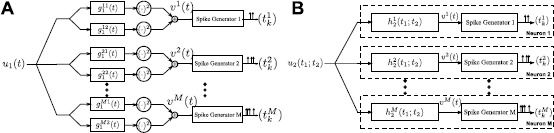
The encoding of temporal stimuli by a neural circuit modeling an ensemble of complex cells. **(A)** The *i*th neuron in the model processes the input $u_{1}(t)$ by two parallel linear filters with impulse responses $g^{i1}_{1}(t)$ and $g^{i2}_{1}(t)$, respectively, followed by squaring. The outputs are summed and then fed into a spike generator. **(B)** An equivalent representation of the encoding circuit in which the DSPs are represented as second-order Volterra kernels

We consider a neural circuit consisting of *M* neurons as shown in Fig. 2A. For the *i*th neuron, input stimulus $u_{1}(t)$ ($u_{1}\in {\mathcal {H}}_{1}$) is first processed by two linear filters with impulse responses $g^{i1}_{1}(t)$ and $g^{i2}_{1}(t)$, the outputs of which are individually squared and then summed together. These processing elements are integral part of the DSP of neuron *i* [18, 19]. The output of the DSP *i*, denoted by $v^{i}(t)$, is then fed into the biological spike generator (BSG) of neuron *i*. The BSG *i* encodes the output of DSP *i* into the spike train $(t^{i} _{k})_{k\in \mathbb{I}^{i}}$. Here $\mathbb{I}^{i}$ is the spike train index set of neuron *i*. We notice the similarity between the overall structure of neural circuits in Figs. 2A and 1. In what follows, we refer to the neurons in the neural circuit in Fig. 2A as complex cells.

The output of the DSP of the *i*th neuron in Fig. 2A amounts to

3 $$ v^{i}(t) = \biggl[ \int_{\mathbb{D}} g^{i1}_{1}(t-s_{1}) u_{1}(s_{1})\,ds _{1} \biggr] ^{2} + \biggl[ \int_{\mathbb{D}} g^{i2}_{1}(t-s_{2}) u_{1}(s _{2})\,ds_{2} \biggr] ^{2} $$

for all $i=1,2,\ldots ,M$.

With

4 $$ h^{i}_{2}(t_{1};t_{2}) = g^{i1}_{1}(t_{1})g^{i1}_{1}(t_{2})+g^{i2} _{1}(t_{1})g^{i2}_{1}(t_{2}), $$

(3) can be rewritten as

5 $$ v^{i}(t) = \int_{\mathbb{D}^{2}} h^{i}_{2}(t-s_{1};t-s_{2}) u_{1}(s _{1}) u_{1}(s_{2}) \,ds_{1}\,ds_{2} , $$

where $h^{i}_{2}(t_{1};t_{2})$ is interpreted as a second-order Volterra kernel [25]. We assume that $h_{2}^{i}(t_{1};t _{2})$ is real, bounded-input bounded-output (BIBO) stable, causal, and of finite memory. The I/O of the neural circuit shown in Fig. 2A can be equivalently outlined as in Fig. 2B, in which each neuron processes the input $u_{1}(t)$ nonlinearly by a second-order kernel $h^{i}_{2}(t_{1};t _{2})$ followed by a BSG.

#### Remark 1

Note that the BSG models the spike generation mechanism of the axon hillock of a biological neuron, whereas the DSP is an equivalent model of processing of the stimuli by a sophisticated neural network that proceeds the spike generation. Therefore, stimulus processing and the spike generation mechanism are naturally separated in the neuron model considered here.

For simplicity, we first formulate the spike generation mechanism of the encoder as an ideal integrate-and-fire (IAF) (point) neuron (see, e.g., [17]). The integration constant, bias, and threshold of the IAF neuron $i=1,2,\ldots ,M$ are denoted by $\kappa^{i}$, $b^{i}$, and $\delta^{i}$, respectively. The mapping of the input amplitude waveform $v^{i}(t)$ into the time sequence $(t^{i}_{k})_{k\in \mathbb{I}^{i} }$ is called the *t*-transform [15]. For the *i*th neuron, the t-transform is given by [15, 16]

6 $$ \int_{t^{i}_{k}}^{t^{i}_{k+1}} v^{i}(t)\,dt = \kappa^{i}\delta^{i} - b ^{i} \bigl(t^{i}_{k+1}-t^{i}_{k} \bigr), \quad k\in \mathbb{I}^{i}. $$

#### Lemma 1

*The encoding of the temporal stimulus* $u_{1}\in {\mathcal {H}}_{1}$ *into the spike train sequence* $(t_{k}^{i})$, $k\in \mathbb{I}^{i}$, $i=1,2,\ldots,M$, *by a neural circuit with complex cells is given in functional form by*

7 $$ \mathcal{T}^{i}_{k} u_{2} = q^{i}_{k}, \quad k\in \mathbb{I}^{i}, i = 1, \ldots , M, $$

*where* *M* *is the total number of neurons*, $n_{i}+1$ *is the number of spikes generated by neuron* *i*, *and* $\mathcal{T}^{i}_{k}: {\mathcal {H}}_{2} \rightarrow {\mathbb {R}}$ *are bounded linear functionals defined by*

8 $$ \mathcal{T}^{i}_{k} u_{2} = \int_{t^{i}_{k}}^{t^{i}_{k+1}} \int_{\mathbb{D}^{2}} h^{i}_{2} (t-s_{1};t-s_{2})u_{2}(s_{1}; s_{2})\,ds_{1}\,ds_{2}\,dt $$

*with* $u_{2}(t_{1};t_{2}) = u_{1}(t_{1})u_{1}(t_{2})$. *Finally*, $q^{i}_{k} = \kappa^{i}\delta^{i} - b^{i} (t^{i}_{k+1}-t^{i}_{k})$.

#### Proof

Relationship (7) follows by replacing the functional form of $v^{i}(t)$ given in (5) in equation (6). □

#### Remark 2

The function $u_{2}(t_{1},t_{2})=u_{1}(t_{1})\cdot u_{1}(t_{2})$ can be interpreted as a nonlinear map of the stimulus $u_{1}$ into $u_{2}$ defined in a higher-dimensional space. The operation performed by the second-order Volterra kernel on $u_{2}$ in (8) is linear. Thus, (7) shows that the encoding of temporal stimuli can be viewed as generalized sampling [18].

The above formalism for encoding stimuli with complex cells can be extended in several ways. First, conductance-based BSGs, such as the Hodgkin–Huxley and Morris–Lecar neuron models, and Izhikevich point neuron models, can be employed [26–29]. The encoding can be similarly formulated as generalized sampling [16]. Second, to capture the stochastic nature of spiking neurons, intrinsic noise can be added into the BSG models. For example, an IAF neuron with random thresholds can be used [15, 30]. It is also natural to consider intrinsic noise in the conductance-based BSGs [19]. For both models, it has been shown that the encoding of stimuli can be viewed as *generalized sampling with noisy measurements* [15, 19], that is, the t-transform is of the form

9 $$ \mathcal{T}^{i}_{k} u_{2} = q^{i}_{k} + \varepsilon^{i}_{k}, \quad k\in \mathbb{I}^{i}, i = 1, \ldots , M, $$

where $\mathcal{T}^{i}_{k}$ are bounded linear functionals defined according to the neuron model of choice, and $\varepsilon^{i}_{k}$ represents random noise in the measurements.

In what follows, we will mainly focus on encoding circuits consisting of complex cells whose spiking mechanism is modeled by a deterministic IAF neuron. The results obtained can be extended to the above two cases, and we will provide examples for both of these.

### Decoding of Temporal Stimuli Encoded by a Population of Complex Cells

Assuming that the spike times $(t_{k}^{i}), k\in \mathbb{I}^{i}$, $i=1,2,\ldots,M$ , are known, by Lemma 1 the neural circuit with complex cells encodes the stimulus via a set of linear functionals acting on $u_{2}$ (see equation (7)). Thus, the reconstruction of $u_{2}$ can *in principle* be obtained by inverting the set of linear equations (7) [18].

#### Theorem 1

*The coefficients of* $u_{2}\in {\mathcal {H}}_{2}$ *in* (2) *satisfy the following system of linear equations*:

10 $$ \boldsymbol{\varXi}\mathbf{d} = \mathbf{q},\quad \textit{where } \boldsymbol{ \varXi } = \bigl[ \bigl(\boldsymbol{\varXi}^{1} \bigr)^{T} , \ldots , \bigl(\boldsymbol{\varXi}^{M} \bigr)^{T} \bigr]^{T} \textit{ and } \mathbf{q} = \bigl[ \bigl( \mathbf{q}^{1} \bigr)^{T} ,\ldots, \bigl(\mathbf{q}^{M} \bigr)^{T} \bigr]^{T} $$

*with* $[ \mathbf{q}^{i} ] _{k} = q^{i}_{k}, [ \mathbf{d} ] _{l_{t_{1}}l_{t_{2}}} = d_{l_{t_{1}}l_{t_{2}}} $, *and*

$$ \bigl[ \boldsymbol{\varXi}^{i} \bigr] _{k; l_{t_{1}} l_{t_{2}}} = \int_{t^{i}_{k}}^{t^{i}_{k+1}} e_{l_{t_{1}}+l_{t_{2}}}(t)\,dt \int_{\mathbb{D}^{2}} h^{i}_{2}(s_{1};s_{2}) e_{-l_{t_{1}}}(s_{1}) e _{-l_{t_{2}}}(s_{2}) \,ds_{1}\,ds_{2}. $$

This result can be obtained by plugging (2) into (7). We refer readers to Theorem 1 in [18] for a detailed proof.

We formulate the reconstruction of $u_{2}$ as the following optimization problem:

11 $$ \hat{u}_{2}(t_{1};t_{2}) = \underset{u_{2} \in {\mathcal {H}}_{2}}{ \operatorname{arg\,min}} \sum _{i=1}^{M}\sum_{k\in \mathbb{I}^{i}} \bigl( \mathcal{T}^{i}_{k} u_{2} - q^{i}_{k} \bigr)^{2}. $$

#### Algorithm 1

The solution to (11) is given by

12 $$ \hat{u}_{2}(t_{1};t_{2}) = \sum _{l_{t_{1}}=-L_{t}}^{L_{t}}\sum_{l_{t_{2}}=-L_{t}}^{L_{t}} \hat{d}_{l_{t_{1}} l_{t_{2}}} e_{l_{t_{1}}}(t_{1}) \cdot e_{l_{t_{2}}}(t _{2}), $$

where $\hat{\mathbf{d}} = [\hat{d}_{{-L_{t}},{-L_{t}}},\ldots , \hat{d}_{{-L_{t}},{L_{t}}}, \ldots , \ldots , \hat{d}_{{L_{t}},{-L _{t}}}, \ldots , \hat{d}_{{L_{t}},{L_{t}}}]^{T}$ is obtained by

13 $$ \hat{\mathbf{d}} = \boldsymbol{\varXi}^{\dagger}\mathbf{q} $$

with ^†^ denoting the pseudoinverse operator.

We note that a necessary condition for perfect recovery is that the total number of spikes exceeds $\operatorname{dim}({\mathcal {H}}_{1})(\operatorname{dim}({\mathcal {H}}_{1})+1)/2+M$ [19]. Therefore, the complexity of the decoding algorithm is of order $\operatorname{dim}({\mathcal {H}}_{1})^{2}$.

Following [18, 19], the decoding algorithm is called a Volterra time decoding machine (Volterra TDM).

### Functional Identification of DSPs of Complex Cells

In this section, we formulate the functional identification of a single complex cell in the neural circuit described in Fig. 2A. We perform *M* experimental trials. In trial *i*, $i=1,\ldots ,M$, we present a controlled stimulus $u^{i}_{1}(t)$ to the cell and observe the spike times $(t^{i}_{k})_{k \in \mathbb{I}^{i}}$. We assume that the cell has a DSP of the form $h_{2}(t_{1};t_{2}) = g^{1}_{1}(t_{1})g^{1}_{1}(t_{2}) + g^{2}_{1}(t _{1})g^{2}_{1}(t_{2})$ and an integrate-and-fire BSG with integration constant, bias, and threshold denoted by $\kappa , b$, and *δ*, respectively. The objective is to functionally identify $h_{2}$ from the knowledge of $u_{1}^{i}$ and the observed spikes $(t^{i}_{k})_{k\in \mathbb{I}^{i}}$, $i=1,\ldots ,M$. This is a standard practice in neurophysiology for inferring the functional form of a component of a sensory system [1].

#### Definition 3

Let $h_{p} \in \mathbb{L}^{1}(\mathbb{D}^{p})$, $p=1,2$, where $\mathbb{L}^{1}$ denotes the space of Lebesgue-integrable functions. The operator $\mathcal{P}_{1}: \mathbb{L}_{1}(\mathbb{D}) \rightarrow {\mathcal {H}}_{1}$ given by

14 $$ (\mathcal{P}_{1} h_{1}) (t) = \int_{\mathbb{D}} h_{1} \bigl(t' \bigr) K_{1} \bigl(t; t' \bigr)\,dt' $$

is called the projection operator from $\mathbb{L}^{1}(\mathbb{D})$ to ${\mathcal {H}}_{1}$. Similarly, the operator $\mathcal{P}_{2}: \mathbb{L} _{1}(\mathbb{D}^{2}) \rightarrow {\mathcal {H}}_{2}$ given by

15 $$ (\mathcal{P}_{2}h_{2}) (t_{1};t_{2}) = \int_{\mathbb{D}^{2}} h_{2} \bigl(t'_{1}; t'_{2} \bigr) K_{2} \bigl(t_{1},t_{2}; t'_{1},t'_{2} \bigr) \,dt'_{1}\,dt'_{2} $$

is called the projection operator from $\mathbb{L}^{1}(\mathbb{D}^{2})$ to ${\mathcal {H}}_{2}$.

Note, that $\mathcal{P}_{1} u_{1}^{i} = u_{1}^{i}$ for $u_{1}^{i} \in {\mathcal {H}}_{1}$. Moreover, for $u_{2}^{i}(t_{1},t_{2}) = u_{1}^{i}(t _{1})u_{1}^{i}(t_{2})$, $\mathcal{P}_{2} u_{2}^{i} = u_{2}^{i}$.

#### Lemma 2

*With* *M* *trials of stimuli* $u^{i}_{2}(t_{1};t_{2}) = u^{i}_{1}(t_{1})u ^{i}_{1}(t_{2})$, $i=1,\ldots ,M$, *presented to a complex cell having DSP* $h_{2}(t_{1},t_{2})$, *we have*

16 $$ \mathcal{L}^{i}_{k} (\mathcal{P}_{2} h_{2}) = q_{k}^{i}, \quad k\in \mathbb{I}^{i}, i = 1,\ldots , M, $$

*where*

17 $$ \mathcal{L}^{i}_{k} (\mathcal{P}_{2} h_{2}) = \int_{t^{i}_{k}}^{t^{i} _{k+1}} \int_{\mathbb{D}^{2}} u^{i}_{2}(t-s_{1}; t-s_{2}) ( \mathcal{P} h_{2}) (t-s_{1};t-s_{2}) \,ds_{1}\,ds_{2}\,dt $$

*and*

18 $$ q_{k}^{i} = \kappa^{i}\delta^{i} - b^{i} \bigl(t^{i}_{k+1}-t^{i}_{k} \bigr). $$

#### Proof

See Appendix 1. □

#### Remark 3

**Fig. 3:**
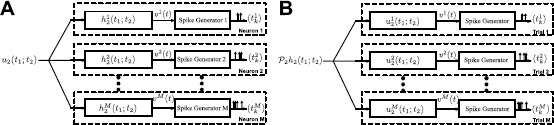
Duality between decoding and identification. **(A)** The stimulus $u_{1}(t)$ is encoded with a population of complex cells. **(B)** The projection of the second-order Volterra DSP of an arbitrary neuron on the input space generates the same spike trains if the impulse responses of the DSPs are the same as the input stimuli in repeated trials

The similarity between equations (7) and (72) suggests that the identification of a complex cell DSP by presenting multiple stimuli is dual to decoding a stimulus encoded by a population of complex cells. This duality is schematically shown in Fig. 3.

#### Theorem 2

*Let* $\mathcal{P}_{2}h_{2} \in {\mathcal {H}}_{2}$ *be of the form*

19 $$ \mathcal{P}_{2}h_{2}(t_{1};t_{2}) = \sum _{l_{t_{1}}=-L_{t}}^{L_{t}}\sum_{l_{t_{2}}=-L_{t}}^{L_{t}} h_{l _{t_{1}} l_{t_{2}}} e_{l_{t_{1}}}(t_{1}) \cdot e_{l_{t_{2}}}(t_{2}) . $$

*Then*, $[ \mathbf{h} ] _{l_{t_{1}}l_{t_{2}}} = h_{l_{t_{1}}l _{t_{2}}}$ *with* $l_{t_{1}} = -L_{t},\ldots ,L_{t}$, $l_{t_{2}} = -L_{t}, \ldots ,L_{t}$, *satisfies the following system of linear equations*:

20 $$ \boldsymbol{\varTheta}\mathbf{h} = \mathbf{q}, $$

*where* $\boldsymbol{\varTheta} = [ (\boldsymbol{\varTheta}^{1})^{T} , \ldots , (\boldsymbol{\varTheta}^{M})^{T} ]^{T}$ *and* $\mathbf{q} = [ (\mathbf{q}^{1})^{T} ,\ldots, ( \mathbf{q}^{M})^{T} ]^{T} $ *with* $[ \mathbf{q}^{i} ] _{k} = q ^{i}_{k}$ *and*

21 $$ \bigl[ \boldsymbol{\varTheta}^{i} \bigr] _{k; l_{t_{1}} l_{t_{2}}} = \int_{t^{i}_{k}}^{t^{i}_{k+1}} e_{l_{t_{1}}+l_{t_{2}}}(t)\,dt \int_{\mathbb{D}^{2}} u^{i}_{2}(s_{1};s _{2}) e_{-l_{t_{1}}}(s_{1}) e_{-l_{t_{2}}}(s_{2}) \,ds_{1}\,ds_{2}. $$

Thus, to identify $\mathcal{P}_{2} h_{2}$, we can follow the same methodology as in Algorithm 1 and formulate the functional identification of $\mathcal{P}_{2} h_{2}$ as

22 $$ \widehat{\mathcal{P}_{2} h_{2}} = \underset{ \mathcal{P}_{2} h_{2} \in {\mathcal {H}}_{2}}{ \operatorname{arg\,min}} \sum_{i=1}^{M} \sum _{k\in \mathbb{I}^{i}} \bigl( \mathcal{L}^{i}_{k}( \mathcal{P}_{2} h _{2}) - q^{i}_{k} \bigr) ^{2}. $$

For a detailed proof, we refer the reader to the proof of Theorem 1 in [18].

#### Algorithm 2

The solution to (22) is given by

23 $$ \widehat{\mathcal{P}_{2}h_{2}}(t_{1};t_{2}) = \sum_{l_{t_{1}}=-L_{t}}^{L_{t}}\sum _{l_{t_{2}}=-L_{t}}^{L_{t}} \hat{h}_{l_{t_{1}} l_{t_{2}}} e_{l_{t_{1}}}(t_{1}) \cdot e_{l_{t_{2}}}(t _{2}), $$

where $\hat{\mathbf{h}} = [\hat{h}_{{-L_{t}},{-L_{t}}},\ldots , \hat{h}_{{-L_{t}},{L_{t}}}, \ldots , \ldots , \hat{h}_{{L_{t}},{-L _{t}}}, \ldots , \hat{h}_{{L_{t}},{L_{t}}}]^{T}$ is obtained by

24 $$ \hat{\mathbf{h}} = \boldsymbol{\varTheta}^{\dagger}\mathbf{q}. $$

The methodology described in Algorithm 2 to identify the nonlinear DSP is called the Volterra channel identification machine (Volterra CIM) [18, 19].

#### Remark 4

Formulating the decoding and identification problems in the tensor product space ${\mathcal {H}}_{2}$ allows the identification of nonlinear processing by solving a set of linear equations. However, due to the increased dimensionality, the algorithm requires for decoding $\mathcal{O} (\operatorname{dim} ( {\mathcal {H}}_{1} ) ^{2} ) $ measurements.

## Low-Rank Decoding and Functional Identification

As shown in Sect. 2.3, a reconstruction of the signal $u_{2}$ is in principle possible by solving a set of linear equations. However, the complexity of the algorithm is prohibitive. We show in this section that an efficient decoding algorithm can be constructed that exploits the structure of encoding circuits with complex cells. Based on the duality between decoding and functional identification, functional identification algorithms that exploit the structure of the DSP of complex cells are presented. These algorithms largely reduce the complexity of decoding of temporal stimuli encoded by an ensemble of complex cells and that of functional identification of their DSPs.

### Low-Rank Decoding of Stimuli

#### Exploiting the Structure of Complex Cell Encoding

In Theorem 1, we introduced a vector notation for the coefficients of $u_{2}$,

25 $$ \mathbf{d} = [d_{{-L_{t}},{-L_{t}}},\ldots ,d_{{-L_{t}},{L_{t}}}, \ldots , \ldots , d_{{L_{t}},{-L_{t}}}, \ldots , d_{{L_{t}},{L_{t}}}]^{T}. $$

We introduce here the matrix notation of the coefficients for $u_{2} \in {\mathcal {H}}_{2}$:

26 $$ \mathbf{D} = \left [ \textstyle\begin{array}{@{}c@{\quad}c@{\quad}c@{}} d_{{-L_{t}},{L_{t}}} & \ldots & d_{{-L_{t}},{-L_{t}}} \\ \vdots & \ddots & \vdots \\ d_{{L_{t}},{L_{t}}} & \ldots & d_{{L_{t}},{-L_{t}}} \end{array}\displaystyle \right ] . $$

We notice the following: (i) since $u_{2}$ is assumed to be real, $\overline{d_{l_{t_{1}},l_{t_{2}}}} = d_{-l_{t_{1}},-l_{t_{2}}}$, and (ii) since $u_{2}(t_{1};t_{2}) = u_{1}(t_{1})u_{1}(t_{2}) = u_{1}(t _{2})u_{1}(t_{1}) = u_{2}(t_{2};t_{1})$, we have $d_{l_{t_{1}},l_{t _{2}}} = d_{l_{t_{2}},l_{t_{1}}} $. These properties imply that **D** is a Hermitian matrix. Moreover, we note that $u_{2}$ in (7) is the ‘outer’ product of the stimuli $u_{1}$, that is,

27 $$ \mathbf{D} = \mathbf{cc}^{H}, $$

where

28 $$ \mathbf{c}= [ c_{-L_{t}}, \ldots , c_{ L_{t}} ] ^{T} $$

are the coefficients of the basis functions of $u_{1}$. Therefore, **D** is a rank-1 Hermitian positive semidefinite matrix. This property will be exploited in stimulus decoding (reconstruction).

##### Theorem 3

*Encoding the stimulus* $u_{1}\in {\mathcal {H}}_{1}$ *with the neural circuit with complex cells given in* (6) *into the spike train sequence* $(t_{k}^{i})$, $k\in \mathbb{I}^{i}$, $i=1,2,\ldots,M$, *satisfies the set of equations*

29 $$ \operatorname{Tr} \bigl( \boldsymbol{\varPhi}^{i}_{k} \mathbf{D} \bigr) = q^{i} _{k},\quad k \in \mathbb{I}^{i}, i = 1,\ldots , M, $$

*where* $\operatorname{Tr}(\cdot )$ *is the trace operator*, **D** *is the rank*-1 *positive semidefinite Hermitian matrix* $\mathbf{D} = \mathbf{c}\mathbf{c}^{H}$, $q^{i}_{k}=\kappa^{i}\delta^{i} - b^{i} (t ^{i}_{k+1}-t^{i}_{k})$ *and* $(\boldsymbol{\varPhi}^{i}_{k})$, $k \in \mathbb{I}^{i}$, $i = 1,\ldots , M$, *are Hermitian matrices with entries in the* $( l_{t_{2}}+L_{t}+1 ) $*th row and* $( l _{t_{1}}+L_{t}+1 ) $*th column given by*

30 $$ \bigl[\boldsymbol{\varPhi}^{i}_{k} \bigr]_{l_{t_{2}}, l_{t_{1}}} = \int_{t^{i}_{k}}^{t^{i}_{k+1}} e_{l_{t_{1}}-l_{t_{2}}}(t)\,dt \int_{\mathbb{D}^{2}} h^{i}_{2}(s_{1};s_{2}) e_{-l_{t_{1}}}(s_{1})e _{l_{t_{2}}}(s_{2}) \,ds_{1}\,ds_{2} . $$

##### Proof

Plugging in the general form of $u_{2}$ in (2) into (8), the left-hand side of (7) amounts to

$$ \sum_{l_{t_{1}}=-L_{t}}^{L_{t}}\sum _{l_{t_{2}}=-L_{t}}^{L_{t}} d_{l _{t_{1}},- l_{t_{2}}} \int_{t^{i}_{k}}^{t^{i}_{k+1}}e_{l_{t_{1}}-l _{t_{2}}}(t)\,dt \int_{\mathbb{D}^{2}} h^{i}_{2}(s_{1};s_{2})e_{-l_{t _{1}}}(s_{1})e_{l_{t_{2}}}(s_{2}) \,ds_{1}\,ds_{2}. $$

It is easy to verify that this expression can be written as

31 $$ \sum_{l_{t_{1}}=-L_{t}}^{L_{t}}\sum _{l_{t_{2}}=-L_{t}}^{L_{t}} d_{l _{t_{1}},- l_{t_{2}}} \bigl[\boldsymbol{ \varPhi}^{i}_{k} \bigr]_{l_{t_{2}}, l _{t_{1}}} = \operatorname{Tr} \bigl(\boldsymbol{\varPhi}^{i}_{k}\mathbf{D} \bigr). $$

Finally, we note that since $h^{i}_{2}$, $i=1,\ldots ,M$, are assumed to be real valued, $(\boldsymbol{\varPhi}^{i}_{k})$, $k\in\mathbb{I}^{i}$, $i=1,\ldots ,M$, are Hermitian. □

##### Remark 5

We note that equation (29) in Theorem 3 and equation (10) in Theorem 1 are the same. These equations represent the t-transform of a complex cell in (rank-1) matrix and vector form, respectively. The (rank-1) matrix representation is made possible by the equality $u_{2}(t_{1}; t_{2}) = u_{1}(t_{1})u_{1}(t_{2})$.

#### Reconstruction Algorithms

Solving the systems of equations (29) and (10) requires at least $\operatorname{dim}({\mathcal {H}}_{1})(\operatorname{dim}({\mathcal {H}}_{1})+1)/2+M$ measurements. Consequently, practical solutions become quickly intractable. Fortunately, the encoded stimulus is of the form $u_{2}(t_{1};t_{2}) = u_{1}(t_{1})u_{2}(t_{2})$. This guarantees that **D** is a rank-1 matrix, and thus the reconstructed stimulus belongs to a small subset of ${\mathcal {H}}_{2}$. Therefore, we can cast the problem of reconstructing temporal stimuli encoded by neural circuits with complex cells as a feasibility problem, that is, finding all positive semidefinite Hermitian matrices that satisfy (29) and have rank 1. As we will demonstrate, the latter condition can be satisfied with substantially fewer measurements.

Recently, there is an increasing interest in low-rank optimizations such as matrix factorization, matrix completion, and rank minimization, both from a theoretical and from a practical standpoint [24, 31, 32]. For example, rank minimization has recently been applied to phase retrieval problems [22].

Our objective here is to find rank-1, positive semidefinite matrices that satisfy the t-transform (29). Since there always exists at least one rank-1 solution, this is equivalent to the following optimization problem [33]:

32 $$ \textstyle\begin{array}{cc} \mbox{minimize} & \operatorname{Rank}{(\mathbf{D})} \\ \mbox{s.t.} & \operatorname{Tr}\bigl( \boldsymbol{\varPhi}^{i}_{k}\mathbf{D}\bigr) = q^{i}_{k}, \quad k \in \mathbb{I}^{i}, i = 1,\ldots , M, \\ & \mathbf{D} \succcurlyeq 0. \end{array} $$

The rank minimization problem in (32) is NP-hard. A well-known heuristic is to relax problem (32) to a trace minimization problem [32], that is, instead of solving (32), we reconstruct $u_{2}$ using Algorithm 3.

##### Algorithm 3

The reconstruction of $u_{2}$ from the spike times generated by the neural circuit with complex cells is given by

33 $$ \hat{u_{2}}(t_{1};t_{2}) = \sum _{l_{t_{1}}=-L_{t}}^{L_{t}}\sum_{l_{t_{2}}=-L_{t}}^{L_{t}} \hat{d}_{l_{t_{1}} l_{t_{2}}} e_{l_{t_{1}}}(t_{1}) \cdot e_{l_{t_{2}}}(t _{2}), $$

where

34 $$ \hat{\mathbf{D}} = \left [ \textstyle\begin{array}{@{}c@{\quad}c@{\quad}c@{}} \hat{d}_{{-L_{t}},{L_{t}}} & \ldots & \hat{d}_{{-L_{t}},{-L_{t}}} \\ \vdots & \ddots & \vdots \\ \hat{d}_{{L_{t}},{L_{t}}} & \ldots & \hat{d}_{{L_{t}},{-L_{t}}} \end{array}\displaystyle \right ] $$

is the solution to the semidefinite programming (SDP) problem

35 $$ \textstyle\begin{array}{cc} \mbox{minimize} & \operatorname{Tr}{(\mathbf{D})} \\ \mbox{s.t.} & \operatorname{Tr}\bigl( \boldsymbol{\varPhi}^{i}_{k}\mathbf{D}\bigr) = q^{i}_{k}, \quad k\in \mathbb{I}^{i}, i = 1,\ldots , M, \\ & \mathbf{D} \succcurlyeq 0. \end{array} $$

When the matrices $(\boldsymbol{\varPhi}^{i}_{k})$, $k\in\mathbb{I}^{i}$, $i = 1,\ldots , M$, satisfy the rank restricted isometry property [24], the trace norm relaxation converges to the true solution of (32), provided that the number of measurements is of order $\mathcal{O} (\operatorname{dim}(\mathcal{H}_{1})\log (\operatorname{dim }( \mathcal{H}_{1} ) ) )$ [24]. These results suggest that stimuli encoded by complex cells can be decoded with a significantly lower number of measurements than that required by Algorithm 1. To investigate this further, we applied the algorithm to decode a large number of stimuli encoded by complex cells while varying the number of measurements (spikes) used by the decoding algorithm. The results show that the number of spikes required to faithfully represent a stimulus by a neural circuits consisting of complex cells is quasilinearly rather than quadratically proportional to the dimension of the stimulus space. These results are presented in the subsequent sections.

The matrix of weights $\hat{\mathbf{D}}$ obtained from the algorithm can be further decomposed to extract the signal $u_{1}$ (up to a sign) as follows.

(i) Perform the eigen-decomposition of $\hat{\mathbf{D}}$. Denote the largest eigenvalue by *λ* and the corresponding eigenvector by **v**. If (35) does not exactly return a rank-1 matrix, then choose the largest eigenvalue and disregard the rest. Let $\mathbf{w} = \sqrt{\lambda }\mathbf{v}$.

(ii) The reconstructed stimulus $\hat{u}_{1}$ is given by (up to a sign)

$$ \hat{u}_{1}(t) = \sum_{l_{t}=-L_{t}}^{L_{t}} \hat{c}_{l_{t}} e_{l_{t}}(t), $$

where

36 $$ \hat{\mathbf{c}} = \textstyle\begin{cases} \mathbf{w}\cdot \frac{\vert [\mathbf{w}]_{L_{t}+1} \vert }{[\mathbf{w}]_{L_{t}+1}} & \mbox{if } [\mathbf{w}]_{L_{t}+1} \neq 0, \\ \mathbf{w} & \mbox{otherwise,} \end{cases} $$

with $\hat{\mathbf{c}}= [ \hat{c}_{ -L_{t}}, \ldots , \hat{c} _{L_{t}} ] ^{T}$, and $[\mathbf{w}]_{L_{t}+1}$ is the $(L_{t}+1)$th entry of **w**, which corresponds to the coefficient $\hat{c}_{0}$.

If $\hat{\mathbf{D}}$ is rank 1, step (i) decomposes $\hat{\mathbf{D}}$ as an ‘outer’ product of a vector and itself (see (27)). The resulting vector **w** differs from the actual coefficient vector of the stimulus $u_{1}$ by up to a complex-valued scaling factor. This factor is corrected in step (ii). Since $u_{1}$ is assumed to be real valued, the ‘DC’ component must be real valued. Therefore, we rotate **w** to remove any imaginary part. In practice, this also ensures $\hat{c}_{-l_{t}} = \overline{ \hat{c}_{l_{t}}}$.

##### Remark 6

Note that we can reconstruct $u_{1}(t)$ up to a sign, since $\mathbf{D} = \mathbf{c}\mathbf{c}^{H}$ and $\mathbf{D} = (- \mathbf{c})(-\mathbf{c}^{H})$ are equally possible. For clarity, in all examples given in this paper, the sign of the recovered stimulus was matched to the original stimulus.

##### Remark 7

Note that (32) can be alternatively solved by replacing the objective with the log-det heuristic [32], that is,

37 $$ \textstyle\begin{array}{cc} \mbox{minimize} & \log \operatorname{det}(\mathbf{D}+\lambda \mathbf{I}) \\ \mbox{s.t.} & \operatorname{Tr}\bigl( \boldsymbol{\varPhi}^{i}_{k}\mathbf{D}\bigr) = q^{i}_{k},\quad k\in \mathbb{I}^{i}, i = 1,\ldots , M, \\ & \mathbf{D} \succcurlyeq 0, \end{array} $$

where $\lambda > 0$ is a small regularization constant. This optimization may further reduce the rank of $\hat{\mathbf{D}}$ when Algorithm 3 fails to progress to an exact rank-1 solution [32].

##### Remark 8

When intrinsic noise is present in the BSG, the encoding of stimuli can be formulated as generalized sampling with noisy measurements. We modify (35) as follows:

38 $$ \textstyle\begin{array}{cc} \mbox{minimize} & \quad \displaystyle \operatorname{Tr} {(\mathbf{D})} + \lambda \Biggl( \sum\limits_{i=1}^{M}\sum\limits _{k\in \mathbb{I}^{i}} \bigl(\operatorname{Tr}\bigl( \boldsymbol{ \varPhi}^{i}_{k} \mathbf{D}\bigr) - q^{i}_{k} \bigr)^{2} \Biggr) \\ \mbox{s.t.} & \quad \displaystyle \mathbf{D} \succcurlyeq 0, \end{array} $$

where *λ* can be chosen based on the noise estimate. Here, the recovered **D** may no longer be rank-1. The largest rank-1 component is used for the reconstruction of stimuli.

Although the SDP in (35) provides an elegant way for relaxing the rank minimization problem, it is limited in practice by the need of large amounts of computer memory for numerical calculations. The optimization problem (32) can also be solved using an alternating minimization scheme [34] as further outlined in Algorithm 4. The alternating minimization approach is more tractable when the dimension of the space is very large. Algorithm 4 uses an initialization step (step 1) that provides an initial iterate whose distance from **D** is bounded. It then alternately solves for the left and right singular vectors of the rank-1 matrix **D** while keeping the other one fixed (step 2). The resulting subproblems admit a straightforward least squares solution, which can be much more efficiently solved than the SDP in Algorithm 3. Moreover, the algorithm is amenable to parallel computation using general purpose graphics processing units (GPGPUs). The latter property makes it even more attractive when the dimension of the stimulus space is large.

##### Algorithm 4

1. Initialize $\hat{\mathbf{c}}_{1}$ and $\hat{\mathbf{c}}_{2}$ to top left and right singular vectors, respectively, of $\sum_{i=1}^{M} \sum_{k\in \mathbb{I}^{i}} q_{k}^{i} {\boldsymbol{\varPhi} _{k}^{i}}$ normalized to $\sqrt{\frac{1}{\sigma }\sum_{i=1}^{M}\sum_{k\in \mathbb{I}^{i}} (q_{k}^{i})^{2}}$, where *σ* is the top singular value of $\sum_{i=1}^{M} \sum_{k\in \mathbb{I}^{i}} q_{k}^{i}{\boldsymbol{\varPhi} _{k}^{i}}$.
2. Solve alternately the following two minimization problems:
  - solve for $\hat{\mathbf{c}}_{1}$ by fixing $\hat{\mathbf{c}}_{2}$
    39 $$ \hat{\mathbf{c}}_{1} = \underset{\mathbf{c}_{1}}{ \operatorname{arg\,min}}\sum _{i=1}^{M} \sum_{k\in \mathbb{I}^{i}} \bigl(\operatorname{Tr} \bigl( \boldsymbol{\varPhi}^{i}_{k} \mathbf{c}_{1} \hat{ \mathbf{c}}_{2}^{H} \bigr) - q^{i}_{k} \bigr)^{2}; $$
  - solve for $\hat{\mathbf{c}}_{2}$ by fixing $\hat{\mathbf{c}}_{1}$
    40 $$ \hat{\mathbf{c}}_{2} = \underset{\mathbf{c}_{2}}{ \operatorname{arg\,min}} \sum _{i=1}^{M} \sum_{k\in \mathbb{I}^{i}} \bigl(\operatorname{Tr} \bigl(\boldsymbol{\varPhi}^{i}_{k} \hat{\mathbf{c}}_{1}\mathbf{c}_{2}^{H} \bigr) - q^{i}_{k} \bigr)^{2} $$
  until $\sum_{i=1}^{M} \sum_{\in \mathbb{I}^{i}} (\operatorname{Tr}(\boldsymbol{\varPhi}^{i}_{k} \hat{\mathbf{c}}_{1} \hat{\mathbf{c}}_{2}^{H} ) - q ^{i}_{k})^{2} \leq \epsilon $, where $\epsilon >0$ is the error tolerance level;
3. compute $\hat{\mathbf{D}} = \hat{\mathbf{c}}_{1} \hat{\mathbf{c}}_{2} ^{H}$.

The matrix $\hat{\mathbf{D}}$ approximates the coefficients of $u_{2} \in {\mathcal {H}}_{2}$ as in (33). We can reconstruct $u_{1}$ using the (appropriately scaled) top eigenvector of $\frac{1}{2} (\hat{\mathbf{D}} + \hat{\mathbf{D}}^{H})$. This can be obtained directly from $\hat{\mathbf{c}}_{1}$ and $\hat{\mathbf{c}} _{2}$ as follows. Let

41 $$ k = \frac{\hat{\mathbf{c}}_{1}^{H} \hat{\mathbf{c}}_{2}- \hat{\mathbf{c}}_{2}^{H} \hat{\mathbf{c}}_{1} + \sqrt{ ( \hat{\mathbf{c}} _{1}^{H} \hat{\mathbf{c}}_{2}-\hat{\mathbf{c}}_{2}^{H} \hat{\mathbf{c}}_{1} ) ^{2} + 4\hat{\mathbf{c}}_{1}^{H} \hat{\mathbf{c}}_{1}\hat{\mathbf{c}}_{2}^{H}\hat{\mathbf{c}}_{2}}}{2 \hat{\mathbf{c}}_{2}^{H}\hat{\mathbf{c}}_{2}} $$

and

42 $$ \mathbf{w} = \sqrt{\frac{1}{2} \hat{\mathbf{c}}_{2}^{H} \hat{\mathbf{c}}_{1} + k \hat{\mathbf{c}}_{2}^{H} \hat{\mathbf{c}}_{2} } \frac{\hat{\mathbf{c}}_{1} + k\hat{\mathbf{c}}_{2}}{\Vert \hat{\mathbf{c}}_{1} + k\hat{\mathbf{c}}_{2} \Vert }. $$

The reconstructed stimulus $\hat{u}_{1}$ is given by (up to a sign)

$$ \hat{u}_{1}(t) = \sum_{l_{t}=-L_{t}}^{L_{t}} \hat{c}_{l_{t}} e_{l_{t}}(t) $$

with $\hat{\mathbf{c}}$ given by equation (36).

We point out that we made the decoding manageable by exploiting the structure of $u_{2}$. Therefore, no constraint is imposed on the form that $h^{i}(t_{1};t_{2})$ takes, and the decoding algorithms can be applied to neural circuits with neurons whose DSPs take the form of any second-order Volterra kernel.

#### Example: Decoding Temporal Stimuli Encoded with a Population of Complex Cells

Here, the neural circuit we consider consists of 19 complex cells. The DSPs of the complex cells are of the form

43 $$ h_{2}^{i}( t_{1}; t_{2}) = g^{i1}_{1} ( t_{1}) g^{i1}_{1}( t_{2}) + g ^{i2}_{1}( t_{1}) g^{i2}_{1}( t_{2}) , $$

where $g^{i1}_{1}(t)$ and $g^{i2}_{1}(t)$ are quadrature pairs of temporal Gabor filters, and $i=1,\ldots ,19$. The Gabor filters are constructed using a dyadic grid of dilations and translations of the mother wavelets. The mother functions are given by

44 $$ g^{1}_{1}(t) = \operatorname{exp} \biggl( - \biggl( \frac{t^{2}}{0.001} \biggr) \biggr) \operatorname{cos} ( 40\pi t ) $$

and

45 $$ g^{2}_{1}(t) = \operatorname{exp} \biggl( - \biggl( \frac{t^{2}}{0.001} \biggr) \biggr) \operatorname{sin} ( 40\pi t ) . $$

The ensemble of Gabor filters spans the frequency range of the input space. The BSG of the complex cells are point IAF neurons with bias $b^{i} = 2$ and integration constant $\kappa^{i} = 1$, $i = 1,\ldots ,M$. These two parameters are kept the same for all stimuli. Different threshold values are chosen for the IAF neurons to vary the total number of spikes, which can be used to evaluate how many measurements are required for perfectly reconstructing the input stimuli.

The domain of the input space ${\mathcal {H}}_{1}$ is $\mathbb{D} = [0,1]\mbox{ [sec]}$, and $L_{t} = 20$, $\varOmega_{t} = 20\cdot 2\pi $ [rad/sec]. Thus, we have $\operatorname{dim}({\mathcal {H}}_{1}) = 41$. The stimuli were generated by randomly choosing their basis coefficients from an i.i.d. Gaussian distribution.

We tested the encoding and subsequent decoding of 6570 stimuli. The total number of spikes produced for each stimulus ranged from 20 to 220. Reconstructions of the stimuli were performed using Algorithm 3, and the SDPs were solved using SDPT3 [35].

**Fig. 4:**
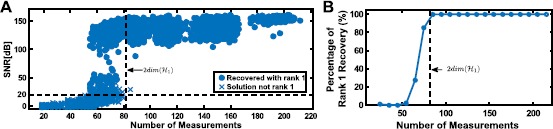
Example of low-rank decoding. **(A)** Effect of number of measurements (spikes) on reconstruction quality. **(B)** Percentage of rank 1 reconstructions

We show the SNR of all reconstructions in the scatter plot of Fig. 4A. Here solid dots represent exact rank 1 solutions (the largest eigenvalue is at least 100 times larger than the sum of the rest of the eigenvalues), and crosses indicate that the trace minimization found a higher rank solution that has a smaller trace. The percentage of exact rank 1 solutions is shown in Fig. 4B. A relatively sharp transition from very low probability of recovery to very high rate of perfect reconstruction can be seen, similar to phase transition phenomena in other sparse recovery algorithms [36]. It can also be seen that the number of measurements that are needed for perfect recovery is substantially lower than the 861 spikes required by decoding based on Theorem 1.

#### Example: IAF Spike Generators with Random Thresholds

Next, for the circuit presented in Sect. 3.1.3, we assumed the IAF neurons to have random thresholds [15]. More specifically, during the interval $[t_{k}^{i}, t_{k+1}^{i})$, the threshold of the *i*th neuron was $\delta_{k}^{i}$, where $\delta_{k} ^{i}$ are i.i.d. Gaussian random variables with mean *δ* and variance $\sigma^{2}$. Since the thresholds are random, the spike times generated by the circuit are no longer deterministic.

**Fig. 5:**
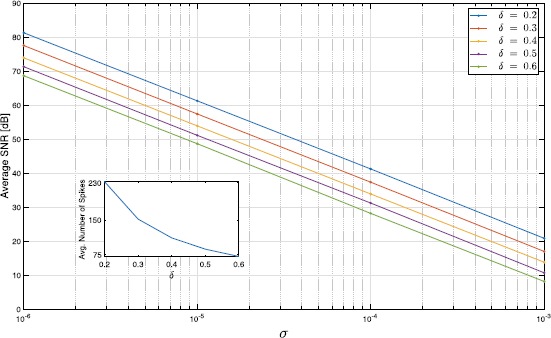
Robust reconstruction of temporal stimuli encoded by complex cells. The BSGs of the complex cells are modeled as ideal IAF neurons with random thresholds. The thresholds of the IAF neurons were independently drawn from $\mathcal{N}(\delta , \sigma^{2})$. The inset shows the average number of spikes generated by the entire circuit for each choice of the threshold *δ*

We chose five different values for *δ* and four different values for *σ*. For each $(\delta , \sigma )$ pair, we presented 50 stimuli to the circuit and subsequently decoded these by solving (38). We found that the SNR of the recovery degrades linearly with $\log (\sigma )$. Figure 5 depicts the average SNR of recovery as a function of *σ* for various *δ*. Note that a lower *δ* corresponds to a higher number of spikes; the inset in the figure provides the average number of spikes produced by the circuit for each *δ*. The results demonstrate that the low-rank decoding algorithm is stable to noise and applicable to non-deterministic encoding paradigms.

#### Example: Hodgkin–Huxley Neurons as Biophysical Spike Generators

**Fig. 6:**
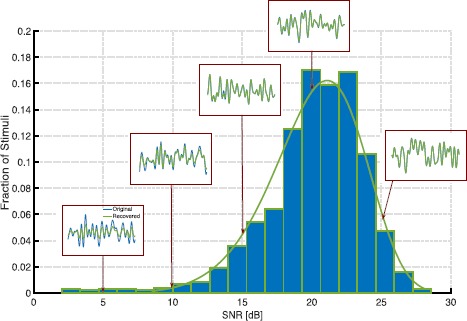
Histogram of reconstruction SNRs of stimuli encoded by complex cells. The BSGs of the complex cells are modeled as Hodgkin–Huxley point neurons. Insets show the original (blue) and recovered (green) stimuli for various SNR values

Here, we evaluate the decoding of stimuli encoded by complex cells with BSGs modeled as Hodgkin–Huxley neurons. The space of the input stimuli and the structure of the DSPs of the neurons are the same as in Sects. 3.1.3 and 3.1.4. However, as the Hodgkin–Huxley point neurons generate significantly more spikes than the IAF neurons considered in the previous examples, we only use here a total of five neurons. Again, the DSPs of these five neurons span the frequency range of the input space. We presented the circuit with 1000 stimuli and subsequently performed their sparse decoding. The average number of spikes generated by the circuit across all stimuli was 215. Figure 6 shows the histogram of the SNRs of the decoded stimuli, with the insets depicting the original and decoded waveforms of a few representative stimuli. These results demonstrate that the low-rank decoding framework presented in this section can also be applied to stimuli encoded with a wide range of spike generators, including the biophysically realistic conductance-based models.

#### Example: Hodgkin–Huxley Neurons with Stochastic Ion Channels

**Fig. 7:**
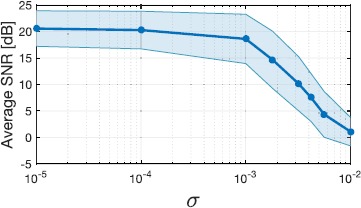
Robust reconstruction of stimuli encoded by complex cells with stochastic ion channels. The BSGs are modeled as Hodgkin–Huxley point neurons with stochastic ion channels. For each noise level *σ*, we set $10\sigma_{1} = \sigma_{2} = \sigma_{3} = \sigma$ where $\sigma^{2}_{i}$, $i=1,2,3$, are the variances of the independent Brownian motion process driving the gating variables *n*, *m*, and *h*, respectively. A larger *σ* represents a higher intrinsic noise strength

Finally, we again consider the same circuit as in Sect. 3.1.5. However, intrinsic ion channel noise is added to the Hodgkin–Huxley point neurons. For a detailed mathematical treatment of Hodgkin–Huxley point neuron with stochastic ion channels, we refer the reader to [19]. Here, independent Brownian motion processes respectively drive each of the gating variables of the Hodgkin–Huxley neuron, that is, *n* (activation of potassium channels), *m* (activation of sodium channels), and *h* (inactivation of sodium channels). The variances of the Brownian motion processes denoted by $\sigma^{2}_{1}$, $\sigma^{2}_{2}$, and $\sigma^{2}_{3}$ were respectively chosen to be $10\sigma_{1} = \sigma_{2} = \sigma_{3} = \sigma $. We presented 50 stimuli to the circuit and repeated the encoding for eight choices of *σ*. For each stimulus presentation, the spike times generated by the circuit were then utilized to recover the stimulus using the sparse reconstruction algorithm. The results are presented in Fig. 7. The points in the figure correspond to the average SNR of the 50 reconstructions for each value of the chosen *σ*, and the shaded area represents their standard deviation. As can be seen from the results, the low-rank decoding framework is robust to intrinsic noise in conductance-based spiking models up to a certain noise level.

### Low-Rank Functional Identification of Complex Cells

#### Duality Between Low-Rank Functional Identification and Decoding

As discussed in Sect. 2.4, the complexity of identification using Algorithm 2 can be prohibitively high. Often, a very large number of stimulus presentation trials are required to fully identify the DSP of biological neurons. To mitigate this, we consider exploiting the structure of the DSP of complex cells as motivated by the tractability of the low rank decoding algorithm.

We consider a single complex cell whose DSP is of the form

46 $$ h_{2}(t_{1};t_{2}) = \sum _{n=1}^{N} g^{n}_{1}(t_{1})g^{n}_{1}(t_{2}), $$

where $g^{n}_{1}(t)$, $n=1,\ldots ,N$, are impulse responses of linear filters, and $N \ll \operatorname{dim}({\mathcal {H}}_{1})$. We note that a complex cell described in Fig. 2A is a particular case of (46) with $N=2$. A natural question here is whether, by assuming such a structure, the functional identification of complex cell DSPs is tractable.

##### Remark 9

It is well known that a second-order Volterra kernel has infinitely many equivalent forms but has a unique symmetric form [25].

We have shown that the low-rank structure of $u_{2}$ leads to a reduction of complexity in the reconstruction of temporal stimuli encoded by an ensemble of complex cells. We also described the duality between decoding and functional identification. If we can show that the functional identification formalism for complex cell DSP is the dual to decoding of low-rank stimuli, then it is straightforward to provide tractable algorithms for identifying $h_{2}(t_{1};t_{2})$ of the form (46).

Since $\mathcal{P}_{1}g^{n}_{1}(t) \in {\mathcal {H}}_{1}, n=1,\ldots ,N$, there is a set of coefficients $(g^{n}_{l_{t}})$, $l_{t} = -L_{t},\ldots,L _{t}$, $n=1,2,\ldots,N$, such that

47 $$ \mathcal{P}_{1}g^{n}_{1}(t) = \sum _{l_{t}=-L_{t}}^{L_{t}} g^{n}_{l _{t}}e_{l_{t}}(t). $$

In what follows, we denote coefficients in vector form as

48 $$ \mathbf{g}^{n} = \bigl[ g^{n}_{-L_{t}}, \ldots , g^{n}_{L_{t}} \bigr] ^{T} . $$

Similarly, we denote the coefficients of $\mathcal{P}_{1}h_{2}(t_{1};t _{2})$ in (19) in matrix form as

49 $$ \mathbf{H} = \left [ \textstyle\begin{array}{@{}c@{\quad}c@{\quad}c@{}} h_{{-L_{t}},{L_{t}}} & \ldots & h_{{-L_{t}},{-L_{t}}} \\ \vdots & \ddots & \vdots \\ h_{{L_{t}},{L_{t}}} & \ldots & h_{{L_{t}},{-L_{t}}} \end{array}\displaystyle \right ] . $$

Then

50 $$ \mathbf{H} = \sum_{n=1}^{N} \mathbf{g}^{n} \bigl(\mathbf{g}^{n} \bigr)^{H}, $$

and thus **H** is a Hermitian positive semidefinite matrix with rank at most *N*.

##### Theorem 4

*By presenting* *M* *trials of stimuli* $u^{i}_{2}(t_{1};t_{2}) = u^{i} _{1}(t_{1})u^{i}_{1}(t_{2}), i=1,\ldots ,M$, *to a complex cell its coefficients satisfy the set of equations*

51 $$ \operatorname{Tr} \bigl( \boldsymbol{\varPsi}^{i}_{k} \mathbf{H} \bigr) = q^{i} _{k}, \quad k\in \mathbb{I}^{i}, i = 1,\ldots , M, $$

*where* $n_{i}+1$, $i=1,\ldots ,M$, *is the number of spikes generated by the complex cell in trial* *i*, **H** *is a Hermitian positive semidefinite matrix with* $\operatorname{rank}(\mathbf{H}) \leq N$ *given by* $\mathbf{H} = \sum_{n=1}^{N} \mathbf{g}^{n}(\mathbf{g}^{n})^{H} $ *with* $\mathbf{g}^{n}= [ g^{n}_{-L_{t}}, \ldots , g^{n}_{L_{t}} ] ^{T}$, $(\boldsymbol{\varPsi}^{i}_{k}), k\in \mathbb{I}^{i}, i = 1, \ldots , M$, *are Hermitian matrices with entry at the* $( l_{t_{2}}+L _{t}+1 ) $*th row and* $( l_{t_{1}}+L_{t}+1 ) $*th column given by*

52 $$ \bigl[\boldsymbol{\varPsi}^{i}_{k} \bigr]_{l_{t_{2}}; l_{t_{1}}} = \int_{t^{i}_{k}}^{t^{i}_{k+1}} e_{l_{t_{1}}-l_{t_{2}}}(t)\,dt \int_{\mathbb{D}^{2}} u_{2}^{i}(s_{1};s_{2})e_{ -l_{t_{1}}}( s_{1})e _{ l_{t_{2}}}( s_{2})\,ds_{1} \,ds_{2}. $$

##### Proof

From Lemma 2 we have

53 $$ \mathcal{L}^{i}_{k} (\mathcal{P}_{2}h_{2}) = q^{i}_{k}, \quad k\in \mathbb{I}^{i}, i=1,\ldots ,M, $$

where

54 $$ \mathcal{L}^{i}_{k} (\mathcal{P}_{2}h_{2}) = \int_{t^{i}_{k}}^{t^{i} _{k+1}} \int_{\mathbb{D}^{2}} u^{i}_{2}(t-s_{1};t-s_{2}) (\mathcal{P} _{2}h_{2}) (s_{1};s_{2}) \,ds_{1}\,ds_{2}\,dt. $$

Equations (51) can be obtained following the steps of the proof of Theorem 3. □

##### Remark 10

**Fig. 8:**
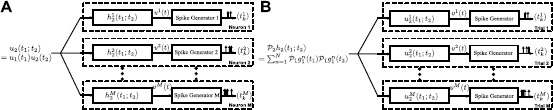
Duality between low-rank decoding and low-rank functional identification. Duality between low-rank decoding of a stimulus encoded by a population of complex cells and low-rank functional identification of complex cells. **(A)** The low-rank decoding algorithm assumes that the encoded stimulus can be written as $u_{2}(t_{1};t_{2})=u_{1}(t_{1})u _{1}(t_{2})$. **(B)** Functional identification of a complex cell assumes that the structure of the DSP is low rank, that is, $\mathcal{P}_{2}h _{2}(t_{1};t_{2}) = \sum_{n=1}^{N} \mathcal{P}_{1}g^{n}_{1}(t_{1}) \mathcal{P}_{1}g^{n}_{1}(t_{2})$

As in Sect. 3.2, we note that the similarity in (51) and (29) indicates the duality between low-rank functional identification of complex cells and low-rank decoding of stimuli encoded by a population of complex cells. The duality is illustrated in Fig. 8.

#### Functional Identification Algorithms

To functionally identify the complex cell DSP, we again employ a rank minimization problem

55 $$ \textstyle\begin{array}{cc} \mbox{minimize} & \mathbf{Rank}{(\mathbf{H})} \\ \mbox{s.t.} & \operatorname{Tr}\bigl( \boldsymbol{\varPsi}^{i}_{k}\mathbf{H}\bigr) = q^{i}_{k}, \quad k\in \mathbb{I}^{i}, i = 1,\ldots , M, \\ & \mathbf{H} \succcurlyeq 0. \end{array} $$

We relax the problem to a trace minimization problem similarly to the approach in the low-rank reconstruction algorithm. Here, the optimal solution will have rank *N*, however. Algorithm 5 is considered for low-rank functional identification of complex cells.

##### Algorithm 5

The functional identification of complex cell DSP from the spike times generated by the neuron in *M* stimulus trials is given by

56 $$ \widehat{\mathcal{P}_{2}h_{2}}(t_{1};t_{2}) = \sum_{l_{t_{1}}=-L_{t}}^{L_{t}}\sum _{l_{t_{2}}=-L_{t}}^{L_{t}} \hat{h}_{l_{t_{1}} l_{t_{2}}} e_{l_{t_{1}}}(t_{1}) \cdot e_{l_{t_{2}}}(t _{2}), $$

where

57 $$ \hat{\mathbf{H}} = \left [ \textstyle\begin{array}{@{}c@{\quad}c@{\quad}c@{}} \hat{h}_{{-L_{t}},{L_{t}}} & \ldots & \hat{h}_{{-L_{t}},{-L_{t}}} \\ \vdots & \ddots & \vdots \\ \hat{h}_{{L_{t}},{L_{t}}} & \ldots & \hat{h}_{{L_{t}},{-L_{t}}} \end{array}\displaystyle \right ] . $$

is the solution to the SDP problem

58 $$ \textstyle\begin{array}{cc} \mbox{minimize} & \operatorname{Tr}{(\mathbf{H})} \\ \mbox{s.t.} & \operatorname{Tr}\bigl( \boldsymbol{\varPsi}^{i}_{k}\mathbf{H}\bigr) = q^{i}_{k}, \quad k\in \mathbb{I}^{i}, i = 1,\ldots , M, \\ & \mathbf{H} \succcurlyeq 0. \end{array} $$

Based on the results for decoding using Algorithm 3 and provided that $h_{2}$ is of the form (46), we intuitively inferred that the number of measurements for the perfect identification of $\mathcal{P}_{2}h_{2}$ is much smaller than $\mathcal{O} (\operatorname{dim}(\mathcal{H}_{1})^{2} )$. We demonstrate that this is the case for a large number of identification examples in the subsequent sections.

This suggests that even if the dimension of the input space becomes large, the functional identification of the DSP of complex cells is still tractable. This result has critical implication for performing neurobiological experiments to functionally identify complex cells. First, it suggests that a much smaller number of stimulus trials is needed for perfect identification. Second, the total number of spikes/measurements that needs to be recorded can be significantly reduced. Both mean that the duration of experiment can be shortened.

##### Remark 11

Note that only the projection of the DSP $h_{2}$ onto the space of input stimuli can be identified.

##### Remark 12

We can use the largest *N* eigenvalues and their respective eigenvectors of $\hat{\mathbf{H}}$ to obtain the projection of individual linear filter components $\widehat{\mathcal{P}_{1}g^{n}_{1}}$, $n=1,\ldots ,N$. However, these components may not directly correspond to $\mathcal{P}_{1}g^{n}_{1} $, $n=1,\ldots ,N$, in that the original projections may not be ‘orthogonal’, whereas the eigenvalue decomposition imposes orthogonality.

As in Algorithm 4, when applied for solving the decoding problem, the rank minimization problem above can be solved using alternating minimization, as further described in Algorithm 6. Here, we solve for the top *N* left and right singular vectors of **H** alternately, where *N* is the rank of the second-order Volterra DSP. We note that the initialization step is akin to running an algorithm very similar to the spike-triggered covariance (STC) algorithm widely used in neuroscience [37–41]. The subsequent steps then improve upon this initial estimate.

##### Algorithm 6

1. Initialize $\hat{\mathbf{H}}_{1}$ and $\hat{\mathbf{H}}_{2}$ to top *N* left and right singular vectors, respectively, of $\sum_{i=1}^{M} \sum_{k=1}^{n_{i}} q_{k}^{i} {\boldsymbol{\varPsi} _{k}^{i}}$ with the *n*th singular vector normalized to $\frac{1}{N}\times \sqrt{\frac{1}{\sigma_{n}}\sum_{i=1}^{M}\sum_{k=1}^{n_{i}} (q_{k}^{i})^{2}}$, where $\sigma_{n}$ is the top *n*th singular value of $\sum_{i=1}^{M}\sum_{k=1}^{n_{i}} q_{k}^{i} {\boldsymbol{\varPsi} _{k}^{i}}$.
2. Solve the following two minimization problems:
  - solve for $\hat{\mathbf{H}}_{1}$ by fixing $\hat{\mathbf{H}}_{2}$
    59 $$ \hat{\mathbf{H}}_{1} = \underset{\mathbf{H}_{1} \in \mathbb{C}^{\operatorname{dim}( \mathcal{H}_{1}) \times N}}{ \operatorname{arg\,min}} \sum _{i=1}^{M} \sum_{k\in \mathbb{I}^{i}} \bigl(\operatorname{Tr} \bigl( \boldsymbol{\varPsi} ^{i}_{k} \mathbf{H}_{1} \hat{\mathbf{H}}_{2}^{H} \bigr) - q^{i}_{k} \bigr)^{2}; $$
  - solve for $\hat{\mathbf{H}}_{2}$ by fixing $\hat{\mathbf{H}}_{1}$
    60 $$ \hat{\mathbf{H}}_{2} = \underset{\mathbf{H}_{2} \in \mathbb{C}^{\operatorname{dim}( \mathcal{H}_{1}) \times N}}{ \operatorname{arg\,min}}\sum _{i=1}^{M} \sum_{k\in \mathbb{I}^{i}} \bigl(\operatorname{Tr} \bigl( \boldsymbol{\varPsi} ^{i}_{k} \hat{\mathbf{H}}_{1} \mathbf{H}_{2}^{H} \bigr) - q^{i}_{k} \bigr)^{2} $$
  until $\sum_{i=1}^{M} \sum_{\in \mathbb{I}^{i}} (\operatorname{Tr}( \boldsymbol{\varPsi}^{i}_{k} \hat{\mathbf{H}}_{1} \hat{\mathbf{H}}_{2}^{H} ) - q ^{i}_{k})^{2} \leq \epsilon $, where $\epsilon >0$ is the error tolerance level;
3. compute $\hat{\mathbf{H}} = \frac{1}{2} ( \hat{\mathbf{H}}_{1} \hat{\mathbf{H}}_{2}^{H} + \hat{\mathbf{H}}_{2} \hat{\mathbf{H}}_{1} ^{H} ) $.

#### Example: Identification of Complex Cell DSPs from Spike Times

In this example, we consider identifying a single complex cell having the following Volterra DSP:

61 $$ h_{2}(t_{1},t_{2}) = g^{1}_{1}(t_{1})g^{1}_{1}(t_{1}) + g^{2}_{1}(t _{1})g^{2}_{1}(t_{2}) , $$

where

62 $$\begin{aligned} g^{1}_{1}(t) &= 50 \exp \biggl( -\frac{(t-0.3)^{2}}{0.002} \biggr) \cos ( 40\pi t ) , \end{aligned}$$

63 $$\begin{aligned} g^{2}_{1}(t) &= 50 \exp \biggl( -\frac{(t-0.3)^{2}}{0.002} \biggr) \sin ( 40\pi t ) . \end{aligned}$$

In repeated trials, we presented to the complex cell 1-second long stimuli chosen from the input space. The domain of the input space ${\mathcal {H}}^{1}_{1}$ is $\mathbb{D} = [0,1]$ (sec) and $L_{t} = 20$, $\varOmega_{t} = 20\cdot 2\pi $ (rad/sec), and thus $\operatorname{dim}({\mathcal {H}}^{1} _{1}) = 41$. The stimuli were generated by independently choosing their basis coefficients from the same Gaussian distribution. We presented a total of 16,600 different stimuli in the repeated trials. We then randomly selected between 30–80 trial subsets such that the total number of spikes in each subset was between 60 and 160. We performed the identification process on each subset using Algorithm 5. The optimization problem was solved using SDPT3.

**Fig. 9:**
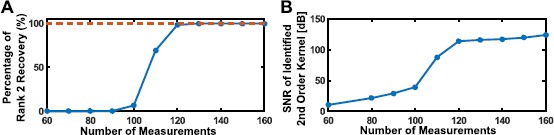
Example of low-rank functional identification. **(A)** Percentage of successful rank-2 recovery in identification. **(B)** Mean SNR of identified second-order DSP kernel

For each instantiation of the identification algorithm, we recorded whether the optimization process resulted in a rank-2 solution and also the SNR of the identified DSP with respect to the original one. For the purpose of demonstration, we binned these results based on number of spikes used into bins of width 10. The percentage of rank-2 solutions is shown in Fig. 9A as a function of number of measurements. The mean SNR is shown in Fig. 9B.

It can be seen from Fig. 9B that the identification algorithm presented here is able to recover the underlying DSP with exceptional accuracy using a reasonable and tractable number of measurements.

### Evaluation of Functional Identification of a Neural Circuit of Complex Cells by Decoding

In Sect. 3.1, we have shown that the sparse decoding algorithm requires much less number of neurons and measurements (spikes) in the reconstruction of stimuli encoded by a neural circuit of complex cells. We have also demonstrated in Sect. 3.2 that the proposed sparse functional identification algorithm enables the identification of complex cells with a tractable number of measurements. Together, the two algorithms afford us tractable functional identification of an entire neural circuit of complex cells that is capable of fully representing stimuli information, in that (i) the size of the neural circuit is tractable and (ii) the requirement for functional identification is tractable.

Decoding of visual stimuli by identified linear filters has previously been considered in [42]. In [17], it was shown that the evaluation of functional identification of an entire neural circuit can be more intuitively performed in the input space by decoding the stimuli with identified circuit parameters. Here, we extend the previous results and apply such evaluation procedure on the sparse decoding and sparse functional identification algorithms. The procedure is described as follows. First, each complex cell is functionally identified using Algorithm 5 or Algorithm 6. Second, novel stimuli are presented to the neural circuit. Third, the spike trains observed are used to reconstruct the encoded novel stimuli by the sparse decoding algorithm, assuming that the circuit parameters take the identified values. Finally, SNR of the reconstruction can be obtained. A high SNR indicates a well-identified circuit, whereas a low number implies that the functional identification of the neural circuit is not of good quality. The latter can be caused by a lack of number of measurements used in functional identification or by a lack of complex cells in the neural circuit.

**Fig. 10:**
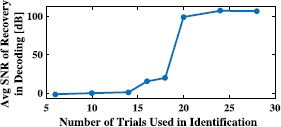
Evaluating identification quality in the input space. Identification quality was evaluated by plotting the average SNR of reconstruction of novel stimuli assumed to be encoded with the identified DSPs

We performed the functional identification of all 19 complex cells in the neural circuit given in the example in Sect. 3.1.3. We first identified all complex cells by presenting to the neural circuit *M* temporal stimuli. We repeated the identification of the entire circuit using eight different values of *M*. We then presented to the same circuit (with the original DSPs as in Sect. 3.1.3) and 100 novel stimuli drawn from the input space and used the spike times generated by the neural circuit to decode the stimuli. In the decoding process, however, we assumed that the DSPs of the set of complex cells are as identified for all eight values of *M*. The mean reconstruction SNR of the 100 stimuli is shown in Fig. 10. As shown, the quality of reconstruction is low until enough trials were used in identification. When more than 19 trials were performed, perfect reconstruction of the entire neural circuit was achieved. The dimension of the stimulus space was 41, and the average number of spikes per neuron used for identification varied from 44 for 6 trials to 202 for 28 trials.

## Low-Rank Decoding and Functional Identification of Complex Cells with Spatio-Temporal Stimuli

The framework introduced in Sect. 3 can be extended to the sparse decoding of spatio-temporal stimuli and the sparse identification of spatio-temporal DSPs of complex cells. Details of the extension to the spatio-temporal case are provided in Appendixes 2–4. In what follows, we present spatio-temporal examples of sparse decoding and identification.

### Low-Rank Decoding of Spatio-Temporal Visual Stimuli

The stimuli $u_{1}$ considered here have *p* spatial dimensions and a single temporal dimension, that is, $u_{1} = u_{1}(x_{1},x_{2},\ldots ,x_{p}, t)$. For simplicity of notation, we use a compact vector notation and denote the spatial variables as $\mathbf{x} = (x_{1},x _{2},\ldots ,x_{p})$. When $p=2$, $u_{1}$ is the usual 2D visual stimulus. The definition of the space of input stimuli is provided in Appendix 2.

The encoding of spatiotemporal stimuli by a population of complex cells and the sparse decoding of spatiotemporal stimuli are formally described in Appendix 3. Note that the output of the DSP of each neuron $i=1,2,\ldots ,M$, can be expressed as

64 $$ v^{i}(t) = \int_{\mathbb{D}^{2}} h^{i}_{2}(\mathbf{x_{1}},t-s_{1}; \mathbf{x_{2}},t-s_{2}) u_{1}(\mathbf{x_{1}},s_{1})u_{1}( \mathbf{x_{2}},s_{2})\,\mathbf{dx_{1}}\,\mathbf{dx_{2}}\,ds_{1}\,ds_{2} , $$

where

65 $$ h^{i}_{2}(\mathbf{x_{1}},t_{1}; \mathbf{x_{2}},t_{2}) = g^{i1}_{1}( \mathbf{x}_{1}, t_{1})g^{i1}_{1}( \mathbf{x}_{2}, t_{2}) + g^{i2}_{1}( \mathbf{x}_{1}, t_{1})g^{i2}_{1}( \mathbf{x}_{2}, t_{2}) $$

has low-rank [18].

In this section, we provide examples that demonstrate the tractability of sparse decoding of spatio-temporal stimuli encoded with complex cells using a small number of spikes.

#### Example: Decoding of 2D Spatio-Temporal Stimuli

We first present an example in which **x** is one-dimensional, that is, $\mathbf{x}=x_{1}$. In this example, our main focus is to illustrate how the number of spikes affects the reconstruction of stimuli encoded by complex cells.

The neural circuit we consider here consists of 62 direction selective complex cells. The low-rank DSPs of the complex cells are of the form

66 $$ h_{2}^{i}(\mathbf{x_{1}}, t_{1}; \mathbf{x_{2}}, t_{2}) = g^{i1}_{1} ( \mathbf{x_{1}}, t_{1}) g^{i1}_{1}( \mathbf{x_{2}}, t_{2}) + g^{i2}_{1}( \mathbf{x_{1}}, t_{1}) g^{i2}_{1}( \mathbf{x_{2}}, t_{2}) , $$

where $g^{i1}_{1}(\mathbf{x}, t)$ and $g^{i2}_{1}(\mathbf{x}, t)$ are quadrature pairs of spatio-temporal Gabor filters, and $i=1,\ldots ,M$. The Gabor filters are constructed from dilations and translations of the mother wavelets on a dyadic grid, where the mother functions are expressed as

67 $$ g^{1}_{1}(\mathbf{x},t) = \operatorname{exp} \biggl( - \biggl( \frac{x_{1} ^{2}}{8} + \frac{t^{2}}{0.001} \biggr) \biggr) \operatorname{cos} ( 1.5x_{1}+20\pi t ) $$

and

68 $$ g^{2}_{1}(\mathbf{x},t) = \operatorname{exp} \biggl( - \biggl( \frac{x_{1} ^{2}}{8} + \frac{t^{2}}{0.001} \biggr) \biggr) \operatorname{sin} ( 1.5x_{1}+20\pi t ) . $$

The BSG of the complex cells are IAF neurons with bias $b^{i} = 10$ and integration constant $\kappa = 1$ for $i = 1,\ldots ,M$. These two parameters are kept the same for all stimuli. Different threshold values are chosen for the IAF neurons to vary the total number of spikes in a larger range to evaluate how many measurements are required for a perfect reconstruction of input stimuli.

The domain of the input space ${\mathcal {H}}^{1}_{1}$ is $\mathbb{D} = [0,32] \times [0,0.4]$ ([a.u.] and [sec], respectively) and $L_{x_{1}} = 6, L _{t} = 4, \varOmega_{x_{1}} = 0.1875\cdot 2\pi , \varOmega_{t} = 10\cdot 2 \pi $ [rad/sec]. Thus, $\operatorname{dim}({\mathcal {H}}^{1}_{1}) = 117$. Stimuli were randomly generated by choosing the basis coefficients to be i.i.d. Gaussian random variables.

We tested the encoding of 1416 stimuli. Each time, a different number of spikes was generated. The reconstruction of stimuli was performed in MATLAB using the extended Algorithm 3, and the SDPs were solved using SDPT3 [35].

**Fig. 11:**
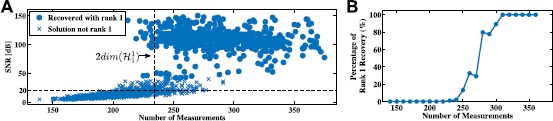
Example of low-rank decoding of spatio-temporal stimuli. **(A)** Effect of number of measurements (spikes) on reconstruction quality. **(B)** Percentage of rank 1 reconstructions

The SNR of all reconstructions is depicted in the scatter plot of Fig. 11A. Here solid dots represent exact rank 1 solutions (largest eigenvalue is at least 100 times larger than the sum of the rest of the eigenvalues), and crosses indicate that the trace minimization found a higher rank solution with a smaller trace. The percentage of exact rank 1 solutions is shown in Fig. 11B. Similar to phase transition phenomena in other sparse recovery algorithms [36], a relatively sharp transition (around 50 spikes) from very low probability of recovery to very high probability of perfect reconstruction can be seen. It can also be seen that the number of measurements that are needed for perfect recovery is substantially lower than the 6965 spikes required by Algorithm 1.

#### Example: Decoding of 3D Spatio-Temporal Stimuli

Next, we present two examples of decoding of spatio-temporal visual stimuli encoded by a population of complex cells. Here, $\mathbf{x} = (x_{1},x_{2})$ and the Volterra DSPs of the complex cells are of the form

69 $$ h_{2}^{i}(\mathbf{x_{1}}, t_{1}; \mathbf{x_{2}}, t_{2}) = g^{i1}_{1} ( \mathbf{x_{1}}, t_{1}) g^{i1}_{1}( \mathbf{x_{2}}, t_{2}) + g^{i2}_{1}( \mathbf{x_{1}}, t_{1}) g^{i2}_{1}( \mathbf{x_{2}}, t_{2}) , $$

where $g^{i1}_{1}(\mathbf{x}, t)$ and $g^{i2}_{1}(\mathbf{x}, t)$ are, for simplicity, quadrature pairs of spatial-only Gabor filters, and $i=1,\ldots ,M$. The Gabor filters are constructed using a dyadic grid of dilations, translations, and rotations of the following pair of mother wavelets [15]:

70 $$ g^{1}_{1}(\mathbf{x},t) = \operatorname{exp} \biggl( - \frac{1}{8} \bigl( 4x_{1}^{2} + 2x_{2}^{2} \bigr) \biggr) \operatorname{cos} ( 2.5x _{1} ) $$

and

71 $$ g^{2}_{1}(\mathbf{x},t) = \operatorname{exp} \biggl( - \frac{1}{8} \bigl( 4x_{1}^{2} + 2x_{2}^{2} \bigr) \biggr) \operatorname{sin} ( 2.5x _{1} ) . $$

The ensemble of Gabor filters forms a frame in the spatial domain of the input space [43].

**Fig. 12:**
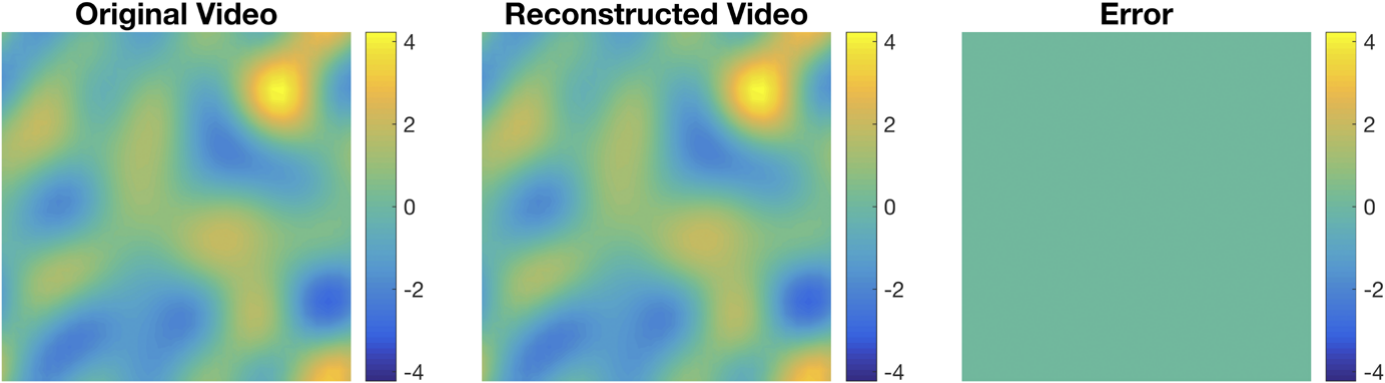
Example of reconstruction of synthesized visual stimuli. A synthetically generated visual stimulus was encoded by 318 Complex Cells that generated some 1374 spikes. A snapshot of the original video is shown on the left. The reconstruction is shown in the middle and the error on the right. SNR 92.8 [dB]. (See also Supplementary Video S1)

For the first example, a 0.4-second-long synthetically generated video sequence is encoded by the neural circuit. The order of the input space was chosen to be $L_{x_{1}} = L_{x_{2}} = 3, L_{t} = 4$. Thus, the dimension of the input space is 441. The input stimulus was created by choosing its basis coefficients to be i.i.d. Gaussian random variables. The stimulus was encoded by a neural circuit consisting of 318 complex cells. A total of 1374 spikes were generated by the encoding circuit. The stimulus was decoded using the extended Algorithm 3. As shown in Fig. 12, the video sequence can be perfectly reconstructed with a fairly small number of spikes (A snapshot of the video is shown; see also Supplementary Video S1 for full video). The SNR of the reconstructed video was 92.8 [dB], thereby reaching almost perfect reconstruction with machine precision. Note that without the reconstruction algorithm employed here, 97,461 measurements would be required from at least 5733 complex cells to achieve perfect reconstruction.

We then performed encoding and subsequent reconstructions of 2-second long natural video sequences that had a resolution of $72 \times 128$ pixels. The videos had temporal bandwidth of 10 [Hz] and spatial bandwidth of 0.375 cycles per pixel. Additionally, the spatial bandwidth was restricted to a circular area to make it isotropically bandlimited. The videos were encoded by a neural circuit consisting of 21,776 complex cells, whose DSPs were modeled as spatial-only quadrature pair of Gabor filters. The Gabor filters formed a frame in the spatial dimension of the space.

The decoding was performed using six NVIDIA P100 GPUs on a single computer node. Despite of their computational power, the amount of memory required by the algorithm for decoding the whole video sequence exceeded the memory capacity of the six GPUs. Therefore, the reconstruction of the entire video was performed by decoding 0.2-second-long segments of the video independently and then stitching them together [16]. The overlap between consecutive segments was 0.1 second. We chose the order of the space to be $L_{x_{1}} = 27, L_{x_{2}} = 48, L_{t} = 3$, and the bandwidth of the space to be $\varOmega_{x_{1}} = \varOmega_{x_{2}} = 0.75\pi$ [rads/pixel] and $\varOmega_{t} = 20\pi$ [rads/s]. We also restricted the spectral lines in the spatial dimension to be inside a circular area instead of a square area as defined in (73), that is, we considered only $l_{x_{1}}$ and $l_{x_{2}}$ that are in the set $\{(l_{x_{1}}, l _{x_{2}}) | l^{2}_{x_{1}}L^{2}_{x_{2}} + l^{2}_{x_{2}}L^{2}_{x_{1}} \leq L^{2}_{x_{1}}L^{2}_{x_{2}}\}$. This allowed the bandwidth of the stimuli to be covered with minimal number of spectral lines [16]. Note that, by the construction of input space, the decoded video must be periodic in time. However, an arbitrary 0.2-second video may not be periodic. Therefore, we chose the decoding space to have a temporal period of 0.3 seconds and retained only the middle 0.2 seconds of the reconstructed segments. The total dimension of the decoding space was 28,413. The extended Algorithm 4 was used in decoding.

**Fig. 13:**
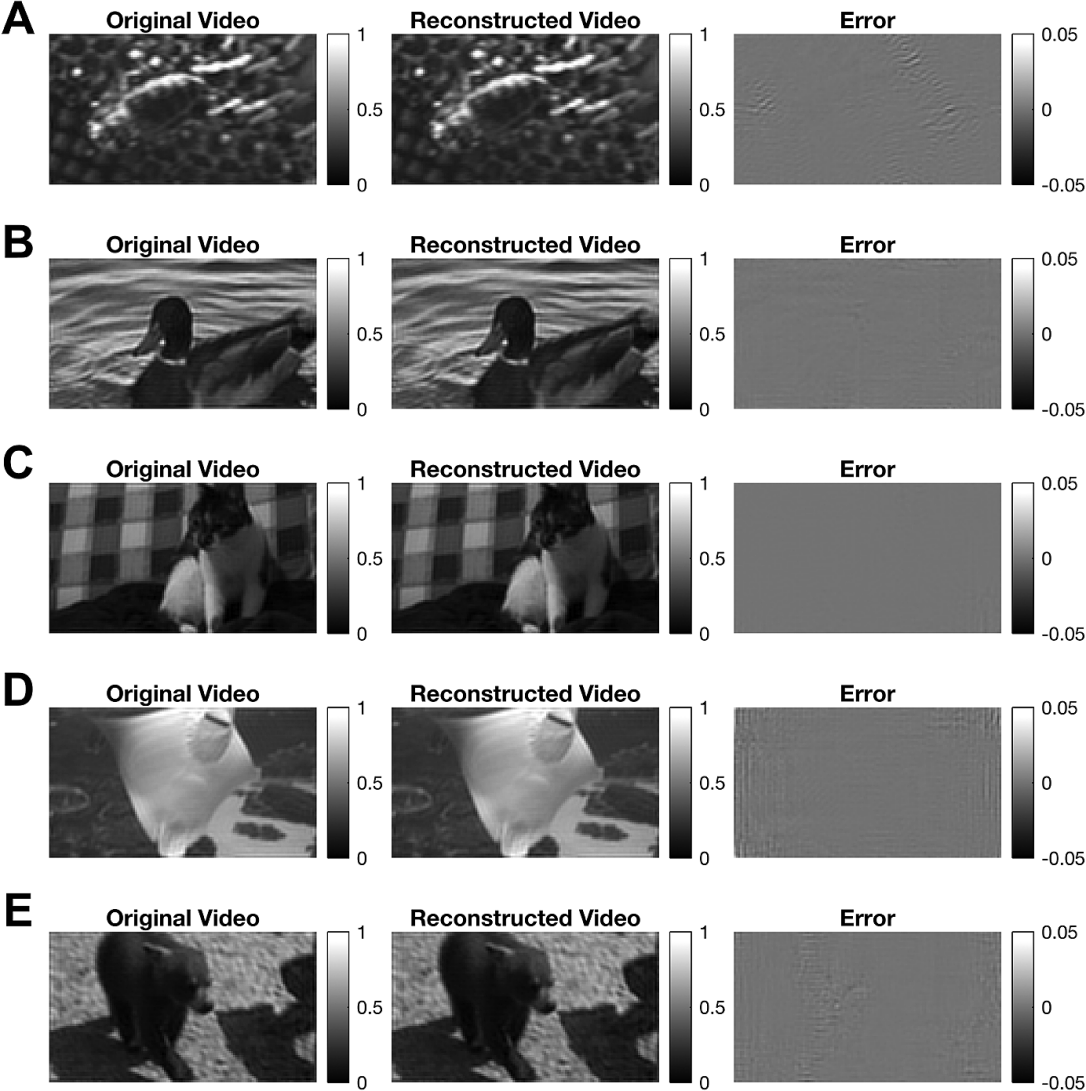
Examples of reconstruction of natural visual stimuli. Snapshots of the original videos encoded by a neural circuit with complex cells are shown on the left. The reconstructions from the spike times are shown in the middle and the error on the right. Note that the color bar indicating the magnitude of the error was set to 10% of the input range. SNR: **(A)** 48.85 [dB]. **(B)** 46.92 [dB]. **(C)** 48.61 [dB]. **(D)** 50.76 [dB]. **(E)** 48.11 [dB]. (See also Supplementary Videos S2–S6)

For the example depicted in Fig. 13A, a total of 980,730 spikes were generated by the neural circuit. About 76,000 to 86,500 measurements were used in reconstructing the video in each time segment. This is approximately 2.67 to 3.04 times of the dimension of the space. In contrast, a total of 403,663,491 measurements would have been required by Algorithm 1 to reconstruct the same video. In Fig. 13A, snapshots of the original video sequence, the reconstructed video sequence and the error are shown (see also Supplementary Video S2) The SNR of the reconstructed video was 48.85 [dB] (the first and last 20 milliseconds were removed from the SNR calculation due to boundary conditions).

Additional examples of reconstructed natural video encoded by the same neural circuit are shown in Fig. 13B–E (see also Supplementary Video S3–S6).

### Low-Rank Functional Identification of Spatio-Temporal Complex Cells

The low-rank functional identification described in Sect. 3.2 can be extended to identify DSPs of spatio-temporal complex cells. The functional identification for the spatio-temporal case is formally described in Appendix 4.

In this section, we first provide an example of identification of spatio-temporal DSPs of complex cells. We then evaluate the identified low-rank spatio-temporal DSPs by decoding novel stimuli encoded with the original neural circuit. The decoding uses the identified filters. Finally, we compare the performance of the low-rank identification methodology with other identification algorithms.

#### Example: Low-Rank Functional Identification of Complex Cell DSP from Spike Times in Response to Spatio-Temporal Stimuli

In this example, we first consider identifying the DSP of a single complex cell in the neural circuit used in Sect. 4.1.1. As a reminder, the neural circuit used in the example in Sect. 4.1.1 encodes spatio-temporal stimuli of the form $u_{1}(x_{1},t)$.

We presented to the population of *M* complex cells 0.4-second stimuli, where *M* varied from 40 to 80. The stimuli were generated by choosing their basis coefficients as i.i.d. Gaussian random variables. For each *M*, we repeated the functional identification process for 200 times, each with different stimuli. Identification was essentially based on the extended Algorithm 3, where the SDPs were again solved by SDPT3.

**Fig. 14:**
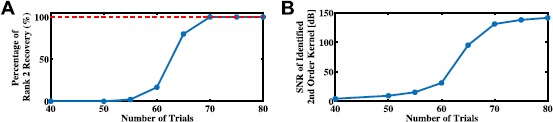
Example of low-rank functional identification of spatio-temporal complex cells. **(A)** Percentage of successful rank 2 recovery in identification. **(B)** Mean SNR of identified second-order DSP kernel

The percentage of rank 2 solutions is shown in Fig. 14A as a function of the number of experimental trials. The mean SNR is shown in Fig. 14B. Figure 14A suggests that if the number of trials is larger than 70, then the solution to the trace minimization coincides with high probability with the rank minimization problem. In contrast, identification of the complex cell DSP using Algorithm 2 would have required at least 407 trials.

It can be easily seen that the identification process does not require a large number of trials to achieve perfect identification, thereby enabling the identification of nonlinear dendritic processing of cells similar in structure to complex cells with a tractable amount of physiological recordings.

#### Example: Evaluation of Functional Identification of Neural Circuit of Complex Cells Using Decoding

We then performed the functional identification of all 62 complex cells in the neural circuit used of the example in Sect. 4.1.1. Here, our goal is to evaluate the identification quality using decoding.

**Fig. 15:**
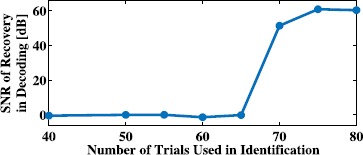
Evaluating identification quality in the input space. SNR of reconstruction of novel stimuli assumed to be encoded with the identified DSPs

We first identified all complex cells by presenting to the neural circuit *M* spatio-temporal stimuli. We also performed the identification of the entire circuit using eight different values of *M*. We then presented to the same circuit 100 novel stimuli drawn from the input space and used the spike times generated by the neural circuit to decode the stimuli. In the decoding process, we assumed that the DSPs of the set of complex cells are as identified for all eight values of *M*. The mean reconstruction SNR of the 100 stimuli is shown in Fig. 15. As shown, the quality of reconstruction was kept at low SNR until enough trials were used in identification. When more than 70 trials were performed, perfect reconstruction was achieved, and thereby the entire neural circuit has been identified with a very high quality.

#### Comparison with STC, GQM, and NIM

We compared the performance of the low-rank functional identification algorithm introduced here with the widely used Spike-Triggered Covariance (STC) algorithm [39]. As in Sect. 4.2.1, a complex cell with a pair of orthogonal Gabor filters was chosen for identification. However, the filters had different norms.

**Fig. 16:**
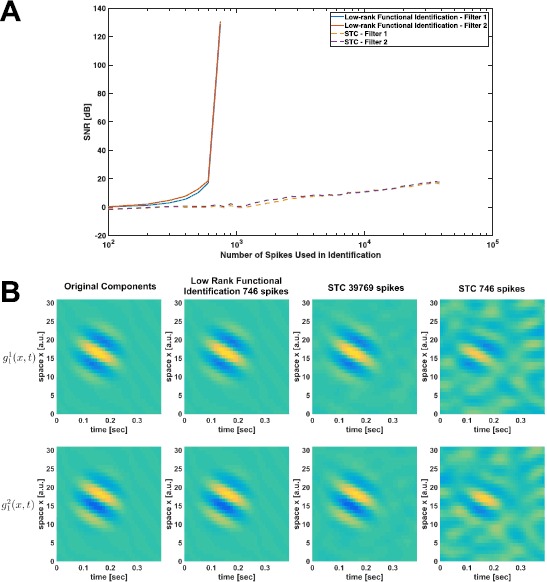
Comparison of the low-rank functional identification with STC. **(A)** SNR of identified quadrature pairs of Gabor filters in a complex cell, as a function of number of spikes used in identification. Low-rank functional identification reaches nearly machine precision with about 746 spikes, which corresponds to about 70 stimulus trials (see also Figure 14). STC reaches about 17 [dB] SNR with ${\sim}30\text{,}000$ spikes. **(B)** Quadrature pair Gabor filters (1st column) identified with low-rank functional identification algorithm with 746 spikes (2nd column, SNR: 128.48 [dB], 130.84 [dB]), and with STC using 39,769 spikes (3rd column, SNR: 16.79 [dB], 17.88 [dB]) and using 746 spikes (4th column, SNR: 0.20 [dB], 0.60 [dB])

Figure 16A shows the quality of identification (SNR) as the number of spikes used in identification increases. Note that the low-rank functional identification algorithm reached perfect identification using only 746 spikes, whereas the performance of the STC algorithm saturated at ∼17 [dB] after almost 40,000 spikes were used. Figure 16B shows the identified individual Gabor filters of the complex cells using both algorithms. The number of spikes used are indicated at the top of each column.

We also evaluated the identification performance of the generalized quadratic model (GQM) [44] and the nonlinear input model (NIM) [45] with quadratic upstream filters to the same example. The results (not shown) were similar to those obtained with the STC algorithm.

We note that whereas the low-rank functional identification algorithm is formulated as nonlinear sampling using TEMs and solved using recent advances in low-rank matrix sensing, the other algorithms tested here rely on moment-based or likelihood-based methods that require a large number of samples to converge.

## Conclusions

In this paper, we presented sparse algorithms for the reconstruction of temporal and spatio-temporal stimuli from spike times generated by neural circuits consisting of complex cells. We formulated the encoding as generalized sampling in a tensor space and exploited the low-rank structure of the stimulus in this space, leading to tractable reconstruction algorithms. For neural circuits consisting of complex cells, this suggests that, in addition to each complex cell extracting visual features, a biologically plausible number of complex cells are capable of faithfully representing visual stimuli. In particular, the examples with natural video sequences provided in this paper demonstrate that neural circuits with nonlinear receptive fields and highly nonlinear spike generating mechanisms are able to faithfully represent natural visual stimuli. The number of spikes that increases just quasi-linearly with the bandwidth or resolution of the stimuli.

Based on duality between sparse decoding and functional identification, we showed that functional identification of complex cells DSPs can be efficiently achieved by exploiting their low-rank structure and using similar algorithms as used in decoding. These algorithms make the functional identification of complex cells tractable, allowing guaranteed high quality identification using a much smaller set of testing stimuli and shorter time duration.

The mathematical treatment presented here, however, is not limited to the complex cells in V1. It can be applied to other neural circuits of interest. For example, early olfactory coding in fruit flies [46] and auditory encoding in grasshoppers [47] have also been shown to have the structure of low-rank DSP kernels. Moreover, the Hassenstein–Reichardt detector [48], a popular model for elementary motion detectors in fruit flies, is also I/O equivalent to low-rank DSP kernels.

### Electronic Supplementary Material

Below are the links to the electronic supplementary material.

Supplementary Video S1. Video demonstrating reconstruction of synthetic visual stimulus encoded by 318 complex cells that generated some 1374 spikes. The original stimulus is shown on the left. The reconstruction is shown in middle and the error on the right. SNR 92.8 [dB] (MP4 2.2 MB)

Supplementary Video S2. Example of reconstructed video encoded by a neural circuit with complex cells. (left column) From top to bottom: original video, reconstructed video and error. (right column) Fourier spectrum of the corresponding frames in the left column. (related to Fig. 13A) (MP4 640 kB)

Supplementary Video S3. Example of reconstructed video encoded by a neural circuit with complex cells. (left column) From top to bottom: original video, reconstructed video and error. (right column) Fourier spectrum of the corresponding frames in the left column. (related to Fig. 13B) (MP4 622 kB)

Supplementary Video S4. Example of reconstructed video encoded by a neural circuit with complex cells. (left column) From top to bottom: original video, reconstructed video and error. (right column) Fourier spectrum of the corresponding frames in the left column. (related to Fig. 13C) (MP4 547 kB)

Supplementary Video S5. Example of reconstructed video encoded by a neural circuit with complex cells. (left column) From top to bottom: original video, reconstructed video and error. (right column) Fourier spectrum of the corresponding frames in the left column. (related to Fig. 13D) (MP4 617 kB)

Supplementary Video S6. Example of reconstructed video encoded by a neural circuit with complex cells. (left column) From top to bottom: original video, reconstructed video and error. (right column) Fourier spectrum of the corresponding frames in the left column. (related to Fig. 13E) (MP4 609 kB)

## Figures and Tables

**Fig. 17:**
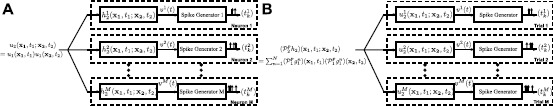
Duality between low-rank decoding and low-rank functional identification for spatio-temporal complex cells. **(A)** decoding of spatio-temporal stimuli encoded by a neural circuit of *M* complex cells and **(B)** functional identification of spatio-temporal complex cells by presenting *M* trials of stimuli

## Appendix 1: Proof of Lemma 2

### Proof

With (18), the t-transform for the *i*th stimulus is given by

$$ \int_{t^{i}_{k}}^{t^{i}_{k+1}} \int_{\mathbb{D}^{2}} h_{2} (t-s_{1};t-s _{2}) u^{i}_{2}(s_{1}; s_{2}) \,ds_{1}\,ds_{2}\,dt = q_{k}^{i}. $$

Since $\mathcal{P}_{2} u_{2}^{i} = u_{2}^{i}$, we have

$$\begin{aligned} \int_{t^{i}_{k}}^{t^{i}_{k+1}} \int_{\mathbb{D}^{2}} h_{2} (t-s_{1};t-s _{2}) \bigl(\mathcal{P}_{2} u^{i}_{2} \bigr) (s_{1}; s_{2})\,ds_{1}\,ds_{2}\,dt &= q _{k}^{i} \end{aligned}$$

or

$$\begin{aligned} \int_{t^{i}_{k}}^{t^{i}_{k+1}} \int_{\mathbb{D}^{2}} \int_{\mathbb{D}^{2}} h_{2} (t-s_{1};t-s_{2})K_{2} \bigl(s_{1},s_{2}; s'_{1},s'_{2} \bigr) u^{i}_{2} \bigl(s'_{1}; s'_{2} \bigr)\,ds'_{1} \,ds'_{2}\,ds_{1}\,ds_{2}\,dt &= q_{k}^{i} \end{aligned}$$

or

$$\begin{aligned} &\int_{t^{i}_{k}}^{t^{i}_{k+1}} \int_{\mathbb{D}^{2}} \int_{\mathbb{D}^{2}} h_{2} (t-s_{1};t-s_{2}) \\ &\quad {}\times K_{2} \bigl(t-s_{1},t-s_{2}; t-s'_{1},t-s'_{2} \bigr)\,ds _{1}\,ds_{2} u^{i}_{2} \bigl(s'_{1}; s'_{2} \bigr) \,ds'_{1}\,ds'_{2}\,dt =q_{k}^{i} \end{aligned}$$

or

$$\begin{aligned} \int_{t^{i}_{k}}^{t^{i}_{k+1}} \int_{\mathbb{D}^{2}} (\mathcal{P} _{2}h_{2}) (t-s_{1};t-s_{2}) u^{i}_{2}(s_{1}; s_{2})\,ds_{1}\,ds_{2}\,dt &= q_{k}^{i}. \end{aligned}$$

Finally, with (17), we obtain

72 $$ \mathcal{L}^{i}_{k} (\mathcal{P}_{2} h_{2}) = q_{k}^{i}, \quad k\in \mathbb{I}^{i}, i = 1,\ldots , M. $$

 □

## Appendix 2: Modeling of Spatio-Temporal Stimuli

### Definition 4

The space of trigonometric polynomials ${\mathcal {H}}_{1}^{p}$ is the Hilbert space of complex-valued functions

73 $$ u_{1}(\mathbf{x},t) = \sum_{\mathbf{l_{x}}} \sum _{l_{t}} c_{ \mathbf{l_{x}}l_{t}}e_{\mathbf{l_{x}}l_{t}}( \mathbf{x},t), $$

where

$$ \begin{aligned} \mathbf{l_{x}}& \in \bigl\{ (l_{x_{1}},l_{x_{2}},\ldots ,l_{x_{p}}) \in \mathbb{Z}^{p} | {-}L_{x_{1}} \leq l_{x_{1}} \leq L_{x_{1}}, -L_{x_{2}} \leq l_{x_{2}} \leq L_{x_{2}}, \ldots , \\ &\quad {}{-}L_{x_{p}} \leq l_{x_{p}} \leq L_{x_{p}} \bigr\} , \\ l_{t}&\in \{k\in \mathbb{Z} | {-}L_{t} \leq k \leq L_{t} \} \end{aligned} $$

over the domain $\mathbb{D}$, where, by abuse of notation, $\mathbb{D} = [0,S_{x_{1}}] \times [0,S_{x_{2}}] \times \cdots \times [0,S_{x_{p}}] \times [0, S_{t}] $ and $S_{t} = \frac{2\pi L_{t}}{ \varOmega_{t}} , S_{x_{1}} = \frac{2\pi L_{x_{1}}}{\varOmega_{x_{1}}} , S_{x_{2}} = \frac{2\pi L_{x_{2}}}{\varOmega_{x_{2}}}, \ldots , S_{x_{p}} = \frac{2\pi L_{x_{p}}}{\varOmega_{x_{p}}}$. In addition, $e_{ \mathbf{l_{x}}l_{t}}(\mathbf{x},t) = e_{\mathbf{l_{x}}}(\mathbf{x})e _{l_{t}}(t)$, where

$$ e_{\mathbf{l_{x}}}(\mathbf{x}) = \frac{1}{\sqrt{\prod_{i=1}^{p} S _{i}}}\operatorname{exp} \bigl( j { \boldsymbol{\omega} _{x}}^{T} \mathbf{x} \bigr) , \quad { \boldsymbol{\omega} _{x}} = \biggl( \frac{l_{x _{1}}\varOmega_{x_{1}}}{L_{x_{1}}}, \frac{l_{x_{2}}\varOmega_{x_{2}}}{L_{x _{2}}},\ldots , \frac{l_{x_{p}}\varOmega_{x_{p}}}{L_{x_{p}}} \biggr) , $$

and

$$ e_{l_{t}}(t) = \frac{1}{\sqrt{S_{t}}}\operatorname{exp} \biggl( \frac{jl _{t}\varOmega_{t}}{L_{t}}t \biggr) . $$

Here $\varOmega_{t}$ denotes the bandwidth, and $L_{t}$ denotes the order of the space in the temporal domain, whereas $\varOmega_{x_{i}}$ and $L_{x_{i}}$ denote the bandwidth and order of the space in the *i*th spatial variable. Stimuli $u_{1}\in {\mathcal {H}}_{1}^{p}$ are periodic with periods $S_{t} , S_{x_{1}}, \ldots , S_{x_{p}}$.

We denote the temporal dimension of ${\mathcal {H}}_{1}^{p}$ by $\operatorname{dim}_{t}({\mathcal {H}}_{1}^{p}) = 2L_{t}+1 $ and the total dimension by $\operatorname{dim}({\mathcal {H}}_{1} ^{p}) = (2L_{t}+1) \prod_{i=1}^{p} (2L_{x_{i}}+1) $.

### Definition 5

The tensor product space ${\mathcal {H}}_{2}^{p} = {\mathcal {H}}_{1}^{p} \otimes {\mathcal {H}}_{1}^{p}$ is the Hilbert space of complex-valued functions

74 $$ u_{2}(\mathbf{x_{1}},t_{1};\mathbf{x_{2}},t_{2}) = \sum_{\mathbf{l_{x_{1}}}}\sum_{l_{t_{1}}} \sum_{\mathbf{l_{x_{2}}}} \sum_{l_{t_{2}}} d_{\mathbf{l_{x_{1}}} l_{t_{1}} \mathbf{l_{x_{2}}} l _{t_{2}}} e_{\mathbf{l_{x_{1}}}}( \mathbf{x_{1}}) e_{l_{t_{1}}}(t _{1}) e_{\mathbf{l_{x_{2}}}}(\mathbf{x_{2}}) e_{l_{t_{2}}}(t _{2}) $$

over the domain $\mathbb{D}^{2}$.

Note that $\operatorname{dim}({\mathcal {H}}_{2}^{p}) = (\operatorname{dim}({\mathcal {H}}_{1}^{p}))^{2}$.

## Appendix 3: Encoding of Spatiotemporal Stimuli with a Population of Complex Cells

We consider again a neural circuit consisting of a population of *M* neurons modeling a population of complex cells as illustrated in Fig. 1. The input to the neural circuit is the spatiotemporal stimulus as defined in Sect. 2.

The input stimulus $u_{1}(\mathbf{x},t)$ to neuron *i* is first processed by two spatio-temporal linear filters whose impulse responses are denoted, by abuse of notation, as $g^{i1}_{1}(\mathbf{x},t)$ and $g^{i2}_{1}(\mathbf{x},t)$, respectively. The output of the linear filters are squared and summed. The sum $v^{i}(t)$, as the output of the DSP, is then fed into the BSG of neuron *i*. The BSG encodes the DSP output into the spike train $(t^{i}_{k})_{k\in \mathbb{I}^{i}}$. Here $\mathbb{I}^{i}$ is the spike train index set of neuron *i*.

Similarly to the temporal case, the neural circuit is equivalent to that shown in Fig. 17A. Here, the output of the DSP for each neuron $i=1,2,\ldots ,M$ can be expressed as

75 $$ v^{i}(t) = \int_{\mathbb{D}^{2}} h^{i}_{2}(\mathbf{x_{1}},t-s_{1}; \mathbf{x_{2}},t-s_{2}) u_{1}(\mathbf{x_{1}},s_{1}) u_{1}( \mathbf{x_{2}},s_{2})\,\mathbf{dx_{1}}\,\mathbf{dx_{2}}\,ds_{1}\,ds_{2}, $$

where

76 $$ h^{i}_{2}(\mathbf{x_{1}},t_{1}; \mathbf{x_{2}},t_{2}) = g^{i1}_{1}( \mathbf{x}_{1}, t_{1})g^{i1}_{1}( \mathbf{x}_{2}, t_{2}) + g^{i2}_{1}( \mathbf{x}_{1}, t_{1})g^{i2}_{1}( \mathbf{x}_{2}, t_{2}) $$

is the low-rank DSP [18]. The encoding of stimulus by the neural circuit with complex cells is a particular case of the low-rank DSP of the form given in (76). When using IAF point neurons as models of the BSGs, we have the following theorem describing the encoding of stimuli.

### Lemma 3

*The encoding of stimulus* $u_{1}\in {\mathcal {H}}^{p}_{1}$ *into the spike train sequence* $(t_{k}^{i}), k\in \mathbb{I}^{i}, i=1,2,\ldots,M$, *by a neural circuit of spatio*-*temporal complex cells is given in functional form by*

77 $$ \mathcal{T}^{i}_{k} u_{2} = q^{i}_{k}, \quad k\in \mathbb{I}^{i}, i = 1, \ldots , M, $$

*where* $\mathcal{T}^{i}_{k}: {\mathcal {H}}_{2}^{p} \rightarrow {\mathbb {R}}$ *are bounded linear functionals defined by*

78 $$ \mathcal{T}^{i}_{k} u_{2} = \int_{t^{i}_{k}}^{t^{i}_{k+1}} \int_{\mathbb{D}^{2}} h^{i}_{2} (\mathbf{x_{1}},t-s_{1}; \mathbf{x_{2}},t-s_{2}) u_{2}(\mathbf{x_{1}},s_{1}; \mathbf{x_{2}}, s _{2})\,\mathbf{dx_{1}}\,\mathbf{dx_{2}}\,ds_{1}\,ds_{2}\,dt $$

*with* $u_{2}(\mathbf{x_{1}},t_{1};\mathbf{x_{2}},t_{2}) = u_{1}( \mathbf{x_{1}},t_{1})u_{1}(\mathbf{x_{2}},t_{2})$. *Finally*, $q^{i}_{k} = \kappa^{i}\delta^{i} - b^{i} (t^{i}_{k+1}-t^{i}_{k})$.

### Proof

As in Lemma 1, the t-transform of the *i*th IAF neuron is given by (6).

Relationship (77) follows after replacing $v^{i}(t)$ given in (75) in equation (6). □

Similarly to Remark 2, equation (77) shows that the encoding of a stimuli by the neural circuit with low-rank DSPs can be viewed as generalized sampling.

By abuse of notation, we denote by **c** the vector representing the coefficients of $u_{1}$ in (73), and **D** as the matrix representing the coefficients of $u_{2}$ in (74). We skip here the detailed entries of **c** and **D** due to the complexity of the indices, but their construction follows closely with (28) and (26), respectively, and $\mathbf{D} = \mathbf{c} \mathbf{c}^{H}$.

### Theorem 5

*Encoding the stimulus* $u_{1}\in {\mathcal {H}}^{p}_{1}$ *with the neural circuit with complex cells given in* (75) *into the spike train sequence* $(t_{k}^{i}), k\in \mathbb{I}^{i}$, $i=1,2,\ldots,M$, *satisfies the set of equations*

79 $$ \operatorname{Tr} \bigl( \boldsymbol{\varPhi}^{i}_{k} \mathbf{D} \bigr) = q^{i} _{k}, \quad k\in \mathbb{I}^{i}, i = 1,\ldots , M, $$

*where* $\mathbf{D} = \mathbf{c}\mathbf{c}^{H}$ *is a rank*-1 *Hermitian matrix*, *and* $(\boldsymbol{\varPhi}^{i}_{k})$, $k \in \mathbb{I} ^{i}, i = 1,\ldots , M$, *are Hermitian matrices*; $[\boldsymbol{\varPhi}^{i}_{k}]_{\mathbf{l_{x_{2}}}l_{t_{2}}; \mathbf{l_{x_{1}}}l _{t_{1}}}$ *denotes the entry at the* $( (l_{t_{2}}+L_{t_{2}}+1)\prod_{i=1}^{p}(L_{x_{i2}}+1)+ \sum_{j=1}^{p}(l_{x_{j2}}+L_{x_{j2}} +1) \prod_{i=1}^{j-1}(2L_{x_{i2}}+1) ) $*th row and the* $( (l_{t_{1}}+L_{t_{1}}+1)\prod_{i=1}^{p}(L_{x_{i1}}+1)+ \sum_{j=1}^{p}(l_{x_{j1}}+L_{x_{j1}} +1) \prod_{i=1}^{j-1}(2L_{x_{i1}}+1) ) $*th column*, *and*

80 $$\begin{aligned} & \bigl[\boldsymbol{\varPhi}^{i}_{k} \bigr]_{\mathbf{l_{x_{2}}}l_{t_{2}}; \mathbf{l_{x_{1}}}l_{t_{1}}} \\ &\quad = \int_{t^{i}_{k}}^{t^{i}_{k+1}} e_{l_{t_{1}}-l_{t_{2}}}(t)\,dt \\ &\qquad \times{} \int_{\mathbb{D}^{2}} h^{i}_{2}(\mathbf{x_{1}}, s_{1};\mathbf{x_{2}},s _{2}) e_{\mathbf{\mathbf{l_{x_{1}}}}, -l_{t_{1}}}( \mathbf{x_{1}}, s _{1})e_{-\mathbf{l_{x_{2}}}, l_{t_{2}}}(\mathbf{x_{2}}, s_{2})\,\mathbf{dx_{1}}\,ds_{1}\, \mathbf{dx_{2}}\,ds_{2}, \end{aligned}$$

*where* $\mathbf{l_{x_{i}}} = (l_{x_{1i}}, l_{x_{2i}}, \ldots , l_{x _{pi}}), i = 1,2$.

### Proof

Plugging $u_{2}$ in the general form (74) into (78), the left-hand side of (77) amounts to

$$\begin{aligned} &\sum_{\mathbf{l_{x_{1}}}} \sum_{l_{t_{1}}} \sum_{\mathbf{l_{x_{2}}}} \sum_{l_{t_{2}}} d_{\mathbf{l_{x_{1}}},l_{t_{1}},-\mathbf{l_{x_{2}}},-l _{t_{2}}} \int_{t^{i}_{k}}^{t^{i}_{k+1}}e_{l_{t_{1}}-l_{t_{2}}}(t)\,dt \\ & \quad {}\times \int_{\mathbb{D}^{2}} h^{i}_{2}(\mathbf{x_{1}},s_{1};x_{2},s _{2})e_{\mathbf{l_{x_{1}}},-l_{t_{1}}}(\mathbf{x_{1}},s_{1})e_{- \mathbf{l_{x_{2}}},l_{t_{2}}}( \mathbf{x_{2}},s_{2})\,\mathbf{dx_{1} \,dx_{2}}\,ds_{1}\,ds_{2}. \end{aligned}$$

It is easy to verify that this expression can be written as

81 $$ \sum_{\mathbf{l_{x_{1}}}} \sum_{l_{t_{1}}} \sum_{\mathbf{l_{x_{2}}}} \sum_{l_{t_{2}}} d_{\mathbf{l_{x_{1}}},l_{t_{1}},-\mathbf{l_{x_{2}}},-l _{t_{2}}} \bigl[\boldsymbol{\varPhi}^{i}_{k} \bigr]_{\mathbf{l_{x_{2}}}l_{t _{2}}; \mathbf{l_{x_{1}}}l_{t_{1}}} = \operatorname{Tr} \bigl( \boldsymbol{ \varPhi}^{i}_{k}\mathbf{D} \bigr), $$

where the $( (l_{t_{1}}+L_{t_{1}}+1)\prod_{i=1}^{p}(L_{x_{i1}}+1)+ \sum_{j=1}^{p}(l_{x_{j1}}+L_{x_{j1}} +1) \prod_{i=1}^{j-1}(2L_{x_{i1}}+1) ) $th row $( (l_{t_{2}}+L_{t_{2}}+1)\prod_{i=1}^{p}(L_{x_{i2}}+1)+ \sum_{j=1}^{p}(l_{x_{j2}}+L_{x_{j2}} +1) \prod_{i=1}^{j-1}(2L_{x_{i2}}+1) ) $th column entry of **D** amounts to $[ \mathbf{D} ] _{\mathbf{l_{x_{1}}}l_{t_{1}} ;\mathbf{l_{x_{2}}}l_{t_{2}}} = d_{ \mathbf{l_{x_{1}}}, l_{t_{1}},- \mathbf{l_{x_{2}}}, -l_{t_{2}}}$.

Since $u_{2}(\mathbf{x_{1}},t_{1};\mathbf{x_{2}},t_{2}) = u_{1}( \mathbf{x_{1}},t_{1})u_{1}(\mathbf{x_{2}},t_{2})$ and $d_{ \mathbf{l_{x_{1}}}, l_{t_{1}},- \mathbf{l_{x_{2}}}, -l_{t_{2}}} = c _{\mathbf{l_{x_{1}}}, l_{t_{1}}} c_{\mathbf{l_{x_{2}}}, l_{t_{2}}} ^{H} $, thereby $\mathbf{D} = \mathbf{c}\mathbf{c}^{H}$. We also note that since $h^{i}_{2}, i=1,\ldots ,M$, are assumed to be real valued, $(\boldsymbol{\varPhi}^{i}_{k}), k\in \mathbb{I}^{i}, i=1,\ldots ,M$, are Hermitian. □

### Low-Rank Decoding of Spatio-Temporal Visual Stimuli

When using an algorithm similar to Algorithm 1 to reconstruct spatio-temporal stimuli encoded by a neural circuit with complex cells, at least $\operatorname{dim}({\mathcal {H}}^{p}_{1}) ( \operatorname{dim}({\mathcal {H}}^{p}_{1})+1 ) /2$ measurements are required. In addition, at least $\operatorname{dim}({\mathcal {H}}^{p}_{1}) ( \operatorname{dim}({\mathcal {H}}^{p}_{1})+1 ) / (4L_{t}+1)$ neurons are required, a number that can become unrealistically high with an increasing dimension of the input space.

With the observation that $\mathbf{D} = \mathbf{cc}^{H}$ is a rank-one matrix, we can apply algorithms similar to those described in Sect. 3.1.2 to recover spatio-temporal stimuli encoded by a population of spiking neurons with low-rank DSPs.

## Appendix 4: Low-Rank Functional Identification of Spatio-Temporal Complex Cells

Similarly to Sect. 3.2, we consider here the identification of low-rank DSP of complex cells from spike times generated when multiple stimulus trials are presented. We first define the projection operators in ${\mathcal {H}}^{p}_{1}$. Then, based on (75), we show that the duality between decoding and functional identification also holds in the spatio-temporal case.

### Definition 6

Let $h_{n} \in \mathbb{L}^{1}(\mathbb{D}^{n}), n=1,2$, where $\mathbb{L}^{1}$ denotes the space of Lebesgue-integrable functions. The operator $\mathcal{P}_{1}^{p}: \mathbb{L}_{1}(\mathbb{D}) \rightarrow {\mathcal {H}}^{p}_{1}$ given by

82 $$ \bigl(\mathcal{P}_{1}^{p} h_{1} \bigr) ( \mathbf{x}, t) = \int_{\mathbb{D}} h_{1} \bigl( \mathbf{x}', t' \bigr) K^{p}_{1} \bigl(\mathbf{x}, t; \mathbf{x}', t' \bigr)\,\mathbf{dx}' \,dt' $$

is called the projection operator from $\mathbb{L}^{1}(\mathbb{D})$ to ${\mathcal {H}}^{p}_{1}$. Similarly, the operator $\mathcal{P}_{2}^{p}: \mathbb{L}_{1}(\mathbb{D}^{2}) \rightarrow {\mathcal {H}}_{2}$ given by

83 $$\begin{aligned} & \bigl(\mathcal{P}_{2}^{p}h_{2} \bigr) ( \mathbf{x_{1}}, t_{1}; \mathbf{x_{2}}, t _{2}) \\ &\quad = \int_{\mathbb{D}^{2}} h_{2} \bigl(\mathbf{x}'_{\mathbf{1}}, t'_{1}; \mathbf{x}'_{\mathbf{2}}, t'_{2} \bigr) K^{p}_{2} \bigl( \mathbf{x_{1}}, \mathbf{x_{2}}, t_{1},t_{2}; \mathbf{x}'_{\mathbf{1}}, \mathbf{x}'_{ \mathbf{2}}, t'_{1},t'_{2} \bigr)\,\mathbf{dx}'_{\mathbf{1}}\,\mathbf{dx}'_{ \mathbf{2}} \,dt'_{1}\,dt'_{2} \end{aligned}$$

is called the projection operator from $\mathbb{L}^{1}(\mathbb{D}^{2})$ to ${\mathcal {H}}_{2}$.

We consider here complex cells whose low-rank DSP can be expressed more generally as

84 $$ h_{2}(\mathbf{x}_{1}, t_{1}; \mathbf{x}_{2}, t_{2}) = \sum_{n=1}^{N} g ^{n}_{1}(\mathbf{x}_{1},t_{1}) g^{n}_{1}(\mathbf{x}_{2},t_{2}), $$

where, by abuse of notation, $g^{n}_{1}(\mathbf{x},t), n= 1,\ldots ,N$, are impulse responses of spatio-temporal linear filters, and $N \ll \operatorname{dim}({\mathcal {H}}^{p}_{1})$. Similarly to the approach we take in Sect. 3.2, this particular structure can be exploited to identify the projection of $h_{2}$ using tractable algorithms.

By abuse of notation, we denote by $\mathbf{g}^{n}$ the vector representing the coefficients of $\mathcal{P}^{p}_{1}g^{n}_{1}$ and by **H** the matrix representing the coefficients of $\mathcal{P} ^{p}_{2}h_{2}$. The detailed entries of $\mathbf{g}^{n}$ and **H** are constructed similarly to (48) and (49), respectively. In addition, we have $\mathbf{H} = \sum_{n=1}^{N} \mathbf{g}^{n}(\mathbf{g}^{n})^{H}$.

### Theorem 6

*By presenting* *M* *trials with stimuli* $u^{i}_{2}(\mathbf{x_{1}}, t _{1}; \mathbf{x_{2}}, t_{2}) = u^{i}_{1}(\mathbf{x_{1}}, t_{1})u^{i} _{1}(\mathbf{x_{2}}, t_{2}), i=1,\ldots ,M$, *to a complex cell and observing the spike trains* $t^{i}_{k}, k\in \mathbb{I}^{i}, i=1,2, \ldots ,M$, *the coefficients of the projections* $\mathcal{P}_{2}^{p}h _{2}$ *of the DSP of the complex cell satisfy the set of equations*

85 $$ \operatorname{Tr} \bigl( \boldsymbol{\varPsi}^{i}_{k} \mathbf{H} \bigr) = q^{i} _{k}, \quad k\in \mathbb{I}^{i}, i = 1,\ldots , M, $$

*where* **H** *is a rank*-*N* *positive semidefinite Hermitian matrix*, *and* $(\boldsymbol{\varPsi}^{i}_{k})$, $k \in \mathbb{I}^{i}$, $i = 1, \ldots , M$, *are Hermitian matrices with the entry at the* $( (l_{t_{2}}+L_{t_{2}}+1)\prod_{i=1}^{p}(L_{x_{i2}}+1)+ \sum_{j=1}^{p}(l_{x_{j2}}+L_{x_{j2}} +1) \prod_{i=1}^{j-1}(2L_{x_{i2}}+1) ) $*th row and the* $( (l_{t_{1}}+L_{t_{1}}+1)\prod_{i=1}^{p}(L_{x_{i1}}+1)+ \sum_{j=1}^{p}(l_{x_{j1}}+L_{x_{j1}} +1) \prod_{i=1}^{j-1}(2L_{x_{i1}}+1) ) $*th column given by*

86 $$\begin{aligned} &\bigl[\boldsymbol{\varPsi}^{i}_{k}\bigr]_{ \mathbf{l_{x_{2}}}l_{t_{2}}; \mathbf{l_{x_{1}}}l_{t_{1}}} \\ &\quad =\int_{t^{i}_{k}}^{t^{i}_{k+1}} e_{l_{t_{1}},-l_{t_{2}}}(t)\,dt \\ &\quad \quad {}\times \int_{\mathbb{D}^{2}} u^{i}_{2}(\mathbf{x_{1}}, s_{1};\mathbf{x_{2}},s _{2}) e_{\mathbf{\mathbf{l_{x_{1}}}}, -l_{t_{1}}}( \mathbf{x_{1}}, s _{1})e_{-\mathbf{l_{x_{2}}}, l_{t_{2}}}(\mathbf{x_{2}}, s_{2})\,\mathbf{dx_{1}}\,ds_{1}\, \mathbf{dx_{2}}\,ds_{2}, \end{aligned}$$

*where* $\mathbf{l_{x_{i}}} = (l_{x_{1i}}, l_{x_{2i}}, \ldots , l_{x_{pi}})$, $i = 1,2$.

### Proof

Essentially similar to the proof of Theorem 4. □

### Remark 13

Theorems 5 and 6 suggest that decoding of spatio-temporal stimuli encoded by a population of complex cells is dual to the functional identification of the DSP of complex cells presented with multiple stimulus trials. This is further illustrated in Fig. 17. Note that in identification only the projection of the complex cell DSP onto the stimulus space can be identified.

Based on Theorem 6, we can formulate functional identification algorithms for complex cell DSPs of the form (84) with a significant reduction in the number of required trials and spikes. The algorithms are similar to those presented in Sect. 3.2.2.

## Abbreviations

BIBO: Bounded-Input Bounded-Output
BSG: Biophysical Spike Generator
CIM: Channel Identification Machine
DSP: Dendritic Stimulus Processor
GPGPU: General Purpose Graphics Processing Unit
GQM: Generalized Quadratic Model
IAF: Integrate-and-Fire
NIM: Nonlinear Input Model
RK: Reproducing Kernel
RKHS: Reproducing Kernel Hilbert Space
SDP: Semidefinite Programming
SNR: Signal-to-Noise Ratio
STC: Spike-Triggered Covariance
TEM: Time Encoding Machine
TDM: Time Decoding Machine
V1: Primary Visual Cortex
